# Antidiabetic Activity of *Gnidia glauca* and *Dioscorea bulbifera*: Potent Amylase and Glucosidase Inhibitors

**DOI:** 10.1155/2012/929051

**Published:** 2011-07-09

**Authors:** Sougata Ghosh, Mehul Ahire, Sumersing Patil, Amit Jabgunde, Meenakshi Bhat Dusane, Bimba N. Joshi, Karishma Pardesi, Sanjay Jachak, Dilip D. Dhavale, Balu A. Chopade

**Affiliations:** ^1^Institute of Bioinformatics and Biotechnology, University of Pune, Pune 411007, India; ^2^Garware Research Centre, Department of Chemistry, University of Pune, Pune 411007, India; ^3^Animal Sciences Division, Agharkar Research Institute, Pune 411004, India; ^4^Department of Microbiology, University of Pune, Pune 411007, India; ^5^Department of Natural Products, National Institute of Pharmaceutical Education and Research, Mohali 160062, India

## Abstract

Diabetes is a metabolic disorder affecting about 220 million people worldwide. One of the most critical complications of diabetes is post-prandial hyper-glycemia (PPHG). Glucosidase inhibitor and **α**-amylase inhibitors are class of compounds that help in managing PPHG. Low-cost herbal treatment is recommended due to their lesser side effect for treatment of diabetes. Two plants with significant traditional therapeutic potential, namely, *Gnidia glauca* and *Dioscorea bulbifera*, were tested for their efficiency to inhibit **α**-amylase and **α**-glucosidase. Stem, leaf, and flower of *G. glauca* and bulb of *D. bulbifera* were sequentially extracted with petroleum ether, ethyl acetate, and methanol as well as separately with 70% ethanol. Petroleum ether extract of flower of *G. glauca* was found to inhibit **α**-amylase significantly (78.56%). Extracts were further tested against crude murine pancreatic, small intestinal, and liver glucosidase enzyme which revealed excellent inhibitory properties. **α**-glucosidase inhibition provided a strong *in vitro* evidence for confirmation of both *G. glauca* and *D. bulbifera* as excellent antidiabetic remedy. This is the first report of its kind that provides a strong biochemical basis for management of type II diabetes using *G. glauca* and *D. bulbifera*. These results provide intense rationale for further *in vivo* and clinical study.

## 1. Introduction

Diabetes mellitus is a multifactorial disease. It is an endocrine and metabolic disorder characterized by chronic hyperglycemia [1]. Multiple biochemical impairments associate with micro- and macrovascular complications which is a major cause of morbidity and death in Diabetes mellitus [2, 3]. With the numbers of people affected by diabetes multiplying worldwide, the disease is taking an ever-increasing proportion of national and international health care strategies [4]. It is projected to become one of the world's main disablers and killers within the next 25 years [5–7]. The affected most are Asia and Africa, where DM rates are expected to rise by two- to threefolds by 2030 [8]. The modern medicines available for management of diabetes exert serious side effects such as hepatotoxicity, abdominal pain, flatulence, diarrhea, and hypoglycemia [9, 10]. Drug resistance to these medicines is also reported after prolonged treatment. Therefore, apart from currently available therapeutic options, many herbal medicines have been recommended for treatment of diabetes [11]. Traditional herbal medicines have been used throughout the world for a range of diabetes [12]. The *α*-glucosidase inhibition significantly decreases post-prandial hyperglycemia (PPHG) in the treatment of type II diabetic (T2DM) patients [13]. Thus, discovery of a suitable inhibitor of glycosidase with minimum side effects poses a challenge in the search for a potent therapeutic agent. The search for improved and safe natural antidiabetic agents is underway, and the World Health Organization has also recommended the development of herbal medicines in this concern [11, 14]. In view of this background, we selected two important medicinal plants, *Gnidia glauca* and *Dioscorea bulbifera* for the same.

*G. glauca *(Family-Thymelaeaceae) is used as traditional phytomedicine for treating sore throat, abdominal pain, wounds, burns, and snake bites [15]. Leaves have been applied to treat the contusions, swellings, back ache, and joint aches [16]. It is considered as a powerful vesicant. It also has agrochemical applications as a molluscicide, insecticide, piscicide, and even larvicidal agents [17–19]. It has been shown that several Gnidia species possess remarkable antineoplastic activity [20]. It is used as an antiviral agent against rabies in Ethiopia [21]. Its antidiabetic properties are not known.

*D. bulbifera* (Family-Dioscoreaceae) possess profound therapeutic potential. It is found throughout the warmer parts of India called as yam or air potato. It is widely used in traditional Indian and Chinese medicine in the treatment of sore throat, gastric cancer and carcinoma of rectum, and goiter [22, 23]. The various extracts of bulbs of the plant have been reported to be antihyperlipidemic [24], antitumor [25], antioxidant [26], anorexiant [27], analgesic and anti-inflammatory [28], plasmid curing [29] and antihyperglycemic [30]. 

It is important to note that there are no reports about the antidiabetic activity of *G. glauca *till date. Similarly, no research has been carried out to identify the targets that can confirm and support the antidiabetic potential of *D. bulbifera*. Hence, we have investigated inhibitory effect of various extracts on commercially available porcine pancreatic *α*-amylase and glucosidases from pancreas, liver, and small intestine of Swiss mice. Furthermore, the inhibitory activity was confirmed against commercially available *α*-glucosidase from *Bacillus stearothermophilus*. HPLC fingerprints of the extracts were developed for 55 min and compared with the standards like gallic acid, quercetin, and diosgenin.

## 2. Methods

### 2.1. Chemicals

*α*-glucosidase and 4-nitrophenyl *α*-D-glucopyranoside were obtained from Sigma Aldrich, USA DNSA (dinitrosalicylic acid) was obtained from SRL Pvt. Ltd. (Mumbai, India). Petroleum ether, ethyl acetate, methanol, ethanol, dipotassium hydrogen phosphate (K_2_HPO_4_), potassium dihydrogen phosphate (KH_2_PO_4_), methanol, sodium potassium tartarate, sodium hydroxide (NaOH), were obtained from Qualigens, Mumbai, India. Porcine pancreatic *α*-amylase and sodium chloride (NaCl) was obtained from HiMedia Laboratories Mumbai, India. Acarbose was obtained from Bayer Pharmaceuticals Pvt. Ltd. (Mumbai, India). All the chemicals and reagents procured were of A.R. grade.

### 2.2. Plant Material

Fresh, leaves, stems, and flowers of *G. glauca* and bulbs of *D. bulbifera* were collected in the month of January from the Western Ghats, Maharashtra, India. Plant materials were identified and authenticated by botanist from National Research Institute of Ayurvedic Sciences, Central Council for Research in Ayurveda and Siddha, Government of Ayush, Ministry of Health and Family Welfare, Department Of India New Delhi, Nehru Garden, Kothrud Pune, 411038. The specimen voucher number provided for the *G. glauca* was 327 and *D. bulbifera* was 860.

### 2.3. Preparation of Plant Extracts

Leaves, stems, and flowers of *G. glauca* were shade dried at room temperature. Bulbs of *D. bulbifera* were chopped into pieces and shade dried. Dried plant materials were subjected to size reduction to a coarse powder by using dry grinder. 100 g of each of the powder was packed into Soxhlet apparatus and extracted successively with petroleum ether, ethyl acetate and methanol at 80°C (yield 2, 8, and 8%, resp.,). 100 g of each powdered plant material was also subjected to a cold extraction with 70% ethanol in distilled water. Petroleum ether, ethyl acetate and methanol extracts were evaporated to dryness under reduced pressure at 4°C in rotary evaporator while ethanolic extract was subjected to lyophilization and were stored in air-tight containers in refrigerator at 4°C. 20 mg dry weight of each crude extract was further reconstituted in 2 mL of 10% dimethyl sulfoxide (DMSO) in distilled water and 1 : 20 dilution of all these extracts were used for *α*-amylase and the glucosidase inhibition assays.

### 2.4. *α*-Amylase Inhibition Assay

Amylase activity was assayed using a modified Bernfeld method (1955) using starch as substrate. 50 *μ*g mL^−1^ (O.D. 0.4 at 280 nm) of porcine pancreatic *α*-amylase was incubated with 1 mg mL^−1^ samples at 37°C for 10 min [31]. One percent starch was used as substrate. *α*-amylase without any extract was used as control. Reducing sugar was estimated using DNSA assay at A 540 nm and the enzyme units were expressed as micro-molar per minute [32]. One unit of enzyme was defined as the amount of enzyme required to liberate 1 *μ*M of maltose under assay conditions. The final inhibition shown by different samples were compared with the standard inhibitor, acarbose [33]. The inhibitory activity was calculated by using the formula

(1) $$\%\;\text{Inhibition}=\frac{\left(\text{A}540_{\text{Control}}-\text{A}540_{\text{Test}}\right)}{\text{A}540_{\text{Control}}}\times 100.$$

### 2.5. Glucosidase Inhibition Assay with Murine Pancreatic, Liver, and Small Intestinal Extracts

10-week-old Swiss male mice weighing 20 gm were procured from National Toxicology Centre, Pune. Entire procedure was carried out with guidelines of Institutional Animal Ethical Committee. The mouse was starved for 12 h. Pancreas, liver, and small intestine tissues were excised and homogenized with 10 mM ice cold phosphate buffer containing 6 mM NaCl (1 : 10 dilution; w/v) and appropriate amount of protease inhibitors. Tissue homogenates were centrifuged for 10 min at 10,000 r.p.m. and the supernatant was taken as a source of enzyme that was diluted so as to get an absorbance of 0.4 (at 280 nm) [34]. Enzyme inhibition assay was carried out as described above. Percentage inhibition of the samples against pancreatic, small intestinal, and liver glucosidases was calculated [35].

### 2.6. *α*-Glucosidase Inhibition Assay

Glucosidase inhibition assay of extracts of the leaves, stem, and flower of *G. glauca* and bulbs of *D. bulbifera* was carried out as per Sanap et al., 2010 [36]. In brief, 0.1 unit/mL of *α*-glucosidase was mixed with each of the samples and incubated for 1 h at 37°C. Enzyme action for *α*-glucosidase was initiated by addition of 2 mM *p*-nitrophenyl-*α*-D-glucopyranoside in 100 mM phosphate buffer of pH 6.8 and stopped by adding 2 mL of 0.1 M Na_2_CO_3_ after an incubation of 10 min at 37°C. *α*-Glucosidase activity was determined by measuring absorbance of the *p*-nitrophenol released from *p*NPG at 420 nm using Shimadzu Spectrophotometer UV-1601. One unit of glucosidase activity is defined as the amount of enzyme that hydrolyzed 1 *μ*M of *p*-nitrophenyl pyranoside per minute under assay condition.

### 2.7. Development of HPLC Fingerprints

A LC-6AD Shimadzu liquid chromatograph system with automated gradient controller, SPD-M20A Photo Diode Array UV-Vis detector and Phenomenex Luna 5 *μ* C-18 column (250 × 4.6 mm ID) was used. 1 mL of HPLC grade methanol was added to 1 mg of sample and sonicated for 10 min followed by centrifugation at 3000 rpm for 15 min. The volume was made upto 10 mL with methanol and the solution was filtered through 0.22 *μ*m filter (Millipore). 20 *μ*L of sample was injected for development of the chromatogram for 55 min. A binary gradient with mobile phase containing: (solvent A) water-acetic acid (0.5% v/v) and (solvent B) acetonitrile: water-acetic acid (99.5–0.5) = 80 : 20 (v/v). The flow-rate was maintained constant at 1 mL/min and the solvent gradient elution program was as follows: 0–10 min, 90% A, 10% B; 10–20 min, 90–80% A, 10–20% B; 20–30 min, 80–60% A, 20–40% B; 30–40 min, 60–40% A, 40–60% B; 40–45 min, 40–30% A, 60–70% B; and 45–50 min, 30–90% A, 70–10% B. 

### 2.8. Statistical Analysis

All values were expressed as mean ± standard error of mean (S.E.M.), *n* = 3, and analyzed for ANOVA and two tailed Student's *t*-test (*P* < 0.05) [37].

## 3. Results

### 3.1. Porcine Pancreatic *α*-Amylase Inhibitory Activity of Plant Extracts

**Figure 1:**
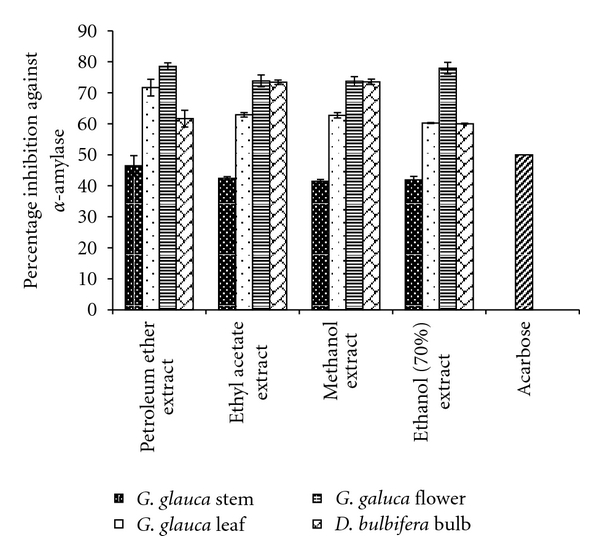
The percent *α*-amylase inhibition by plant extracts. Acarbose is taken as standard inhibitor. The data is indicated as the mean ± SEM; [*n* = 3].

*In vitro* *α*-amylase inhibitory studies demonstrated that the extracts of both *G. glauca *as well as *D. bulbifera *had inhibitory activity. Porcine pancreatic *α*-amylase with 0.29 Umin^−1^ was taken as 100% enzymatic activity. Significant inhibition was exhibited by petroleum ether extract of leaf (IC_50_ = 34.88 *μ*g/mL), and flower (IC_50_ = 31.82 *μ*g/mL) while *D. bulbifera* bulb showed 61.65% (Figure 1). Ethanolic extract of flower exhibited 77.93% inhibition with pure porcine *α*-amylase. Even ethyl acetate (IC_50_ = 33.84 *μ*g/mL), and methanol (IC_50_ = 33.92 *μ*g/mL) extract of flower showed very high inhibition. A significant inhibition of 73.39% was exhibited by ethyl acetate extract of *D. bulbifera* bulbs as well. Both ethyl acetate, and methanol extracts of leaf showed a considerable inhibition of 62.91 and 62.75% against *α*-amylase. Methanol and 70% ethanol extracts of *D. bulbifera* showed considerable inhibition as well. Thus, a significant inhibition was observed with extracts of flower of *G. glauca *and bulbs of* D. bulbifera *bulbs; whereas the extracts of stem showed inhibition lower than both leaf and stem. 

### 3.2. Murine Pancreatic Glucosidases Inhibitory Activity of Plant Extracts

Murine pancreatic enzyme activity exhibiting 0.24 Umin^−1^ was taken as 100% enzymatic activity. Petroleum ether extract of *D. bulbifera* bulb exhibited 22.23% inhibition against murine pancreatic glucosidase, whereas *G. glauca* showed comparatively moderate inhibition of 16.18, 15.92, and 16.37% with stem, leaf, and flower, respectively. In case of ethyl acetate as well *D. bulbifera* bulb was found to be potent inhibitor showing an inhibition of 23.59%. *G. glauca* showed comparable inhibition with each of stem, leaf, and flower. Similar trend was observed in case of methanol and 70% ethanol extract where *D. bulbifera* showed 26% and 18.13%, respectively. Methanol extract of *D. bulbifera* showed significant difference with *P* < 0.05 as compared with other extracts (Table 1).

### 3.3. Murine Small Intestinal Glucosidases Inhibitory Activity of Plant Extracts

Murine small intestinal glucosidase with 0.12 Umin^−1^ was taken as 100% enzymatic activity. Petroleum ether extract of *D. bulbifera* bulb (IC_50_ = 33.62 *μ*g/mL) showed a maximum inhibition of 74.36% that was found to be more potent as compared to acarbose (IC_50_ = 48.79 *μ*g/mL). Petroleum ether extract of *G. glauca* flower showed a maximum inhibition of 57.84%. Ethyl acetate (IC_50_ = 43.22 *μ*g/mL), methanol (IC_50_ = 43.47 *μ*g/mL), and 70% ethanol extract (IC_50_ = 40.4 *μ*g/mL) of *G. glauca* flower showed inhibition comparable to acarbose (Table 2). Ethyl acetate extract of *D. bulbifera* bulb was found to be having an inhibition percentage of 51.41 which was significant as compared to both methanol and 70% ethanol extracts. 

### 3.4. Murine Liver Glucosidases Inhibitory Activity of Plant Extracts

Murine liver glucosidase with 0.75 Umin^−1^ was taken as 100% enzymatic activity. *D. bulbifera* bulb showed a maximum inhibition of 73.36% amongst the petroleum ether extracts while ethyl acetate extract of stem of *G. glauca* showed a potent inhibition of 80%. Methanol extract of *G. glauca* leaf (IC_50_ = 32.61 *μ*g/mL) showed excellent inhibition that was found to be most significant with *P* < 0.05 as compared with other plant extracts as well as acarbose (Table 3). Amongst the 70% ethanol extracts, *D. bulbifera* exhibited a maximum inhibition up to 53.3%. 

### 3.5. *α*-Glucosidase Inhibitory Activity of Plant Extracts

**Figure 2:**
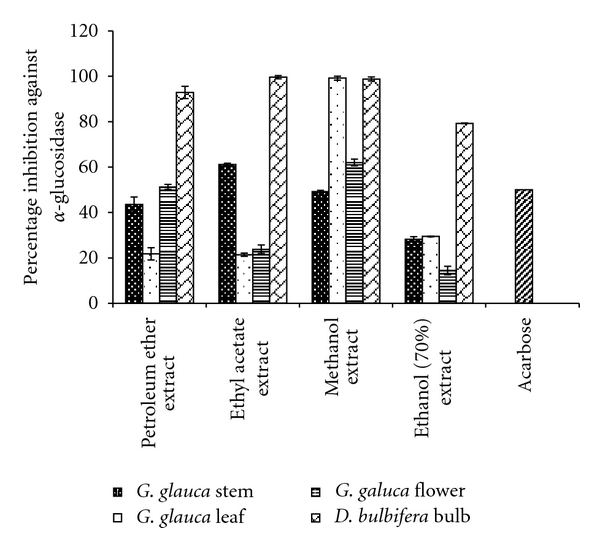
The percent *α*-glucosidase inhibition by plant extracts. Acarbose is taken as standard inhibitor. The data is indicated as the mean ± SEM; [*n* = 3].

0.1 Umin^−1^ of *α*-glucosidase was taken as 100% enzymatic activity. Petroleum ether extract of *D. bulbifera* showed strong inhibitory potential with a percentage inhibition of 92.87% as compared to acarbose. Stem, leaf, and flower of *G. glauca* showed moderate inhibition of 43.54, 21.77 and 51.23%, respectively (Figure 2). Ethyl acetate extract of *D. bulbifera* bulb was found to be the strongest inhibitor showing an inhibition as high as 99.6%. Similarly, methanol and 70% ethanol extracts of *D. bulbifera* bulbs exhibited an inhibition of 98.81 and 79.27% which were most significant as compared to others. 

### 3.6. HPLC Fingerprinting

**Figure 3:**
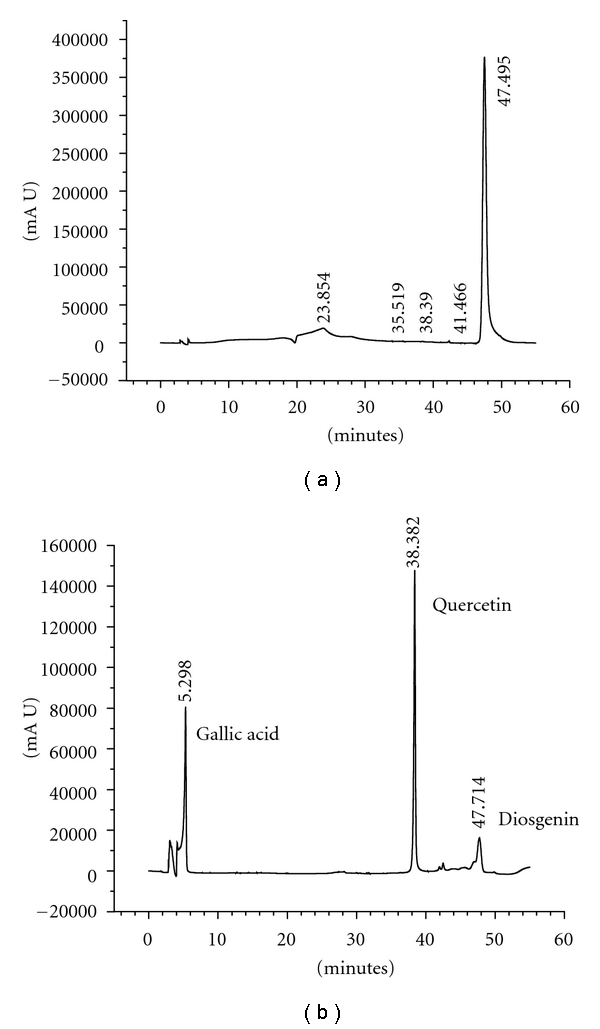
A representative HPLC fingerprint of plant extract (a) *D. bulbifera* methanol extract and (b) HPLC chromatogram of a standard solution containing three marker compounds.

In this study an HPLC method was developed to achieve shorter runtime (55 min) with gradual increase of organic phase (acetonitrile). Gallic acid, quercetin, and diosgenin were used as standards. HPLC fingerprinting showed the high content of diosgenin in the methanol extract of *D. bulbifera* bulb. A binary gradient system consisting of water-acetonitrile-acetic acid as mobile phase was able to separate the compounds in the extracts (Figure 3). The developed HPLC method was applied for detect of the marker compounds and to assess the number of unidentified peaks that indicated the variability in the phytochemical profile of the plant materials. The overlay HPLC fingerprints (See figure s21-24 is supplementary material available online at doi: 10.1155/2012/929051) were used to assess the sample similarity against a generated reference chromatogram. In case of *D. bulbifera* the variability was observed mostly in case of ethyl acetate and methanol extracts. In case of *G. glauca* leaf the maximum diversity in the phytochemistry was characterized by twenty five peaks with different Rt values. Similarly, twenty-two peaks in the methanolic extract of *G. glauca* flower also notably indicated the presence of relatively high amount of phytochemicals of polar nature. Significant number of peaks in the ethyl acetate extract showed the dominance of the probable flavonoids or terpenoid compounds in both *D. bulbifera *and *G. glauca.* The chromatogram also showed several other unidentified peaks as shown in Table 4.

## 4. Discussion

Herbal extracts and herbal formulations, used in the Ayurvedic literarture, have recently been reviewed and have gained importance for the control of T2DM. They are being used directly or indirectly for the preparation of many modern drugs [12]. Although *G. glauca* and *D. bulbifera* have been used for thousands of years as ingredients in Ayurvedic and Chinese traditional medicine, they have not gained much importance as medicines and one of the factors being lack of specific standards being prescribed for herbal medicines and supportive animal/clinical trials [38]. In the present study, we investigated both the plants for their possible significance in control of T2DM by glucosidase inhibitory activity. Plants have wide array of phytochemicals ranging from both nonpolar to polar. Thus, plant materials were sequentially extracted to ensure complete extraction of all non-polar as well as polar components and thereby inclusion of all components in the screening study. Plants are known to produce a large variety of glucosidase inhibitors that provides protection against insects and microbial pathogens [39, 40]. Therefore, plant extracts were analyzed for *α*-amylase inhibitory activity. Pancreatic and intestinal glucosidases are the key enzymes of dietary carbohydrate digestion and inhibitors of these enzymes may be effective in retarding glucose absorption to suppress PPHG. In T2DM, excessive hepatic glycogenolysis and gluconeogenesis is associated with decreased utilization of glucose by tissues being the fundamental mechanism underlying hyperglycemia [41]. The liver glucosidase inhibitors inhibit *α*-1,6-glucosidase of glycogen-debranching enzymes in the liver and reduce the glycogenolytic rate which increases the accumulation of glycogen stores in the liver [42, 43]. Inhibition of these enzyme systems decreases the current blood glucose levels in diabetic patient (as a short-term effect) and shows a small reduction in hemoglobin A1_c_ level (as a long-term effect). 

Our investigation provides the first evidence of antidiabetic property of extracts of *G. glauca*. On the other hand,* D. bulbifera* is a comparatively well-studied plant system due to its anticancer and antioxidant activity [22, 26]. It is widely used in Asia as a rich and sustainable source of carbohydrate. Traditionally, it has gained importance for its antiobese and antihyperglycemic properties. However, there is no scientific evidence for this fact and hereby our investigation is a novel approach to identify the targets involved for lowering the glycemic index and the control of post-prandial hyper-glycemia. In our study, for the first time a detailed biochemical basis of the antidiabetic activity is presented that involves the inhibition of not only the pure *α*-amylase and glucosidase as well as the crude murine enzymes which are the key players of the T2DM. Recently, a preliminary study has shown the antihyperglycemic and antihyperlipidemic activity of *D. bulbifera* on Wistar rats [30]. Hence, there is a need to find out the biochemical basis of lowering of glycemic index *in vitro*.

**Figure 4:**
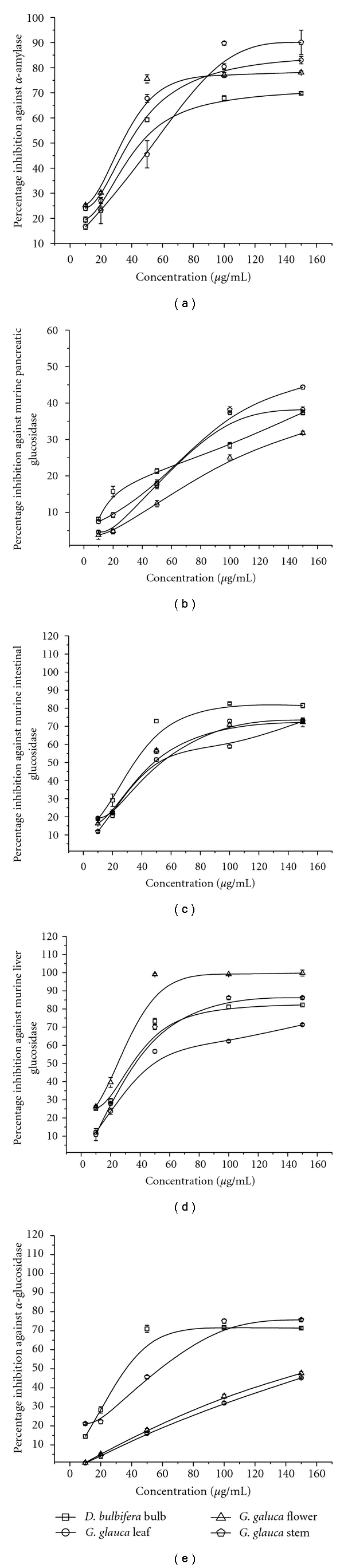
Representative dose response curves for plant extracts. (a) Petroleum ether extracts against porcine pancreatic *α*-amylase; (b) ethyl acetate extracts against murine pancreatic glucosidase; (c) petroleum ether extracts against murine small intestinal glucosidase; (d) petroleum ether extracts against murine liver glucosidase: (e) ethyl acetate extracts against *α*-glucosidase.

Plant extracts are known to be potent *α*-amlase inhibitors due to their rich phenolic content that bind to the reactive sites of enzymes, thus altering its catalytic activity [44]. It has been suggested that the mechanism of inhibition of *α*-amylase may occur through the direct blockage of the active centre at several subsites of the enzyme as also suggested for other inhibitors [45]. A significant variability in the levels of enzyme inhibition between the parts of the same plant evident in the dose response curves (Figure 4) suggests the phytochemical diversity among the parts resulting in variation in the antidiabetic property. Dose response curves for all extracts against, *α*-amylase, murine pancreatic, intestinal, liver glucosidases, and *α*-glucosidase are given in the supplementary information. Our study revealed that petroleum ether extract of *G. glauca* flower (78.56%) and methanolic extract of *D. bulbifera* bulbs (73.54%) were potent *α*-amylase inhibitors. Various herbal extracts like *Bougainvillea spectabilis* and *Trigonella foenum-graecum* seeds are known as  *α*-amylase inhibitors showing 29.43% and 59%, respectively [35, 46]. Thus, *G. glauca* and *D. bulbifera* exhibited comparatively more efficient *α*-amylase inhibition.

The inhibition observed for extracts of* D. bulbifera* bulbs against murine pancreatic glucosidase was found to be comparable as earlier reports on other Indian medicinal plants such as *Linum usitatissimum *methanolic extract [35]. Similarly, leaf extract of *Murraya koenigii* and *Azadirachta indica *are reported to show 43.71% and 41.7% inhibition against murine small intestinal glucosidase, respectively. We found that, the methanolic extract of both *G. glauca* and *D. bulbifera* were found to be superior showing an inhibition at a range of 50–58%. Petroleum ether of *D. bulbifera* bulbs exhibited an inhibition of 73.3% against murine liver glucosidase which was more potent as compared to the maximum inhibition percent of *Bougainvillea spectabilis*,* Ocimum tenuiflorum*, and *Syzygium cumini* as reported earlier. However, extracts of *G. glauca* were found to have superior inhibitory property, particularly petroleum ether extract of flower that exhibited an inhibition in the range of 90–99% while methanol extract of leaf showed highest inhibition (IC_50_ = 25.2 *μ*g/mL) against murine liver glucosidase. Ethanolic extract of *Andrographis paniculata* was reported to inhibit *α*-glucosidase up to 89% only at significantly high concentration of 62.5 mg/mL [47]. However, methanolic extract of *G. glauca* leaf and ethyl acetate extract of *D. bulbifera* showed an inhibition of 99.19 and 99.6%, respectively, at considerably low concentrations (66.65 *μ*g/mL). Moreover, the strong inhibition by methanol extract of flower (IC_50_ = 53.74 *μ*g/mL) against the pure *α*-glucosidase from *Bacillus stearothermophilus* confirmed the antidiabetic property of *G. glauca* as well as its variability on the basis of the parts of plant selected. HPLC fingerprint indicated the presence of diosgenin in the methanolic extracts of *D. bulbifera* bulbs. The maximum number of peaks in the ethyl acetate fractions might be due to the rich flavonoids and terpenoid content in the plant materials. 

We, for the first time, have explored *G. glauca* and *D. bulbifera* as potential glycosidase inhibitors. Presently, we have identified the targets of the extracts being the *α*-amylase and *α*-glucosidase as well as pancreatic, intestinal, and liver enzymes that are principally responsible for hyperglycemia in diabetes. They can further be studied and used as dietary supplement for controlling PPHG in type II diabetes.

## 5. Conclusions

*G. glauca* and *D. bulbifera* show significant inhibition with porcine pancreatic amylase and crude murine glucosidase as well as pure *α*-glucosidase. This may have beneficial effects in managing type II diabetes mellitus and could be used as effective herbal formulation in combinational therapy which can be taken up in further studies.

## Supplementary Material

Dose response curves of petroleum ether, ethyl acetate, methanol and 70% ethanol extract of *D. bulbifera* and *G. glauca* against porcine pancreatic *α*-amylase, crude murine pancreatic, small intestinal, liver glucosidase and **α**-glucosidase are available in the supplementary material. Graphical data for HPLC fingerprinting of all the extracts of *D. bulbifera* and *G. glauca* are presented here.

## Figures and Tables

**Table 1:** Percent murine pancreatic glucosidase inhibition by plant extracts.

Acarbose [40.89 ± 1.03]	% Glucosidase inhibitory activity			
	Petroleum ether	Ethyl acetate	Methanol	Ethanol [70%]
*G. glauca*				
Stem	16.81 ± 0.25	19.32 ± 1.64	16.37 ± 1.35	13.12 ± 1.96
Leaf	15.92 ± 1.9	19.76 ± 1.94	18.76 ± 1.88	14.3 ± 1.03
Flower	16.37 ± 2.6	13.71 ± 2.11	18.87 ± 0.89	16.07 ± 2.48
*D. bulbifera*				
Bulb	22.23 ± 2.71	23.59 ± 0.71	26.1 ± 0.87	18.13 ± 0.15

The data is indicated as the mean ± SEM; [*n* = 3].

**Table 2:** Percent murine small intestinal glucosidase inhibition by plant extracts.

Acarbose [51.23 ± 0.89]	% Glucosidase inhibitory activity			
	Petroleum ether	Ethyl acetate	Methanol	Ethanol [70%]
*G. glauca*				
Stem	57.18 ± 3.75	55.55 ± 2.43	52.28 ± 3.59	47.38 ± 2.84
Leaf	52.61 ± 1.27	50 ± 3.53	49.34 ± 2.01	42.81 ± 3.28
Flower	57.84 ± 2.92	56.84 ± 1.44	57.51 ± 1.81	51.23 ± 0.81
*D. bulbifera*				
Bulb	74.36 ± 2.72	51.41 ± 0.75	50.24 ± 0.83	43.54 ± 0.18

The data is indicated as the mean ± SEM; [*n* = 3].

**Table 3:** Percent murine liver glucosidase inhibition by plant extracts.

Acarbose [37.33 ± 0.69]	% Glucosidase inhibitory activity			
	Petroleum ether	Ethyl acetate	Methanol	Ethanol [70%]
*G. glauca*				
Stem	70 ± 3.28	80 ± 0.56	70 ± 0.59	66.67 ± 1.09
Leaf	56.66 ± 2.71	80 ± 0.71	76.66 ± 0.87	73.33 ± 0.15
Flower	99 ± 1.12	93.33 ± 1.9	93.34 ± 1.5	90 ± 1.81
*D. bulbifera*				
Bulb	73.3 ± 2.73	40 ± 0.74	43.3 ± 0.81	53.3 ± 0.17

The data is indicated as the mean ± SEM; [*n* = 3].

**Table 4:** The retention time of chromatographic peak in the chromatogram of extracts of both *G. glauca* and *D. bulbifera* along with standards.

Peak no.	Retention time of plant extracts																Standard		
	*1*	*2*	*3*	*4*	*5*	*6*	*7*	*8*	*9*	*10*	*11*	12	13	14	15	16	Gallic acid	Quercetin	Diosgenin
*1*	*1.* *67* *5*	*34* *.9* *77*	*1.* *82* *7*	*23* *.8* *54*	*23* *.8* *59*	*23* *.9* *64*	*25* *.* *54* *2*	*24* *.6* *25*	*46* *.8* *4*	*1.* *47* *9*	*1.* *4* *82*	*1.* *83* *1*	*46* *.5* *71*	*19* *.2* *98*	*19* *.3* *13*	*19* *.3* *13*	*5* *.2* *98*	*38* *.3* *82*	*47* *.* *71* *4*
*2*	*1.* *7* *83*	*37* *.6* *82*	*17* *.4* *51*	*35* *.5* *19*	*47* *.3* *77*	*27* *.7* *98*	*28* *.* *34* *2*	*27* *.8* *91*	*47* *.4* *52*	*7.* *18* *9*	*1.* *78* *1*	*28* *.3* *52*	*47* *.2* *82*	*30* *.0* *98*	*30* *.* *19* *8*	*30* *.0* *98*	—	—	—
*3*	*32* *.5* *26*	*38* *.* *46* *3*	*23* *.9* *69*	*38* *.3* *9*	—	*30* *.0* *52*	*28* *.5* *01*	*28* *.3* *53*	—	*28* *.0* *41*	*1.* *8* *67*	*28* *.6* *95*	—	*30* *.4* *5*	*34* *.3* *15*	*30* *.4* *52*	—	—	—
*4*	*38* *.* *46* *6*	*39* *.* *77* *3*	*38* *.3* *68*	*4* *1.* *46* *6*	—	*30* *.4* *41*	*29* *.7* *84*	*29* *.4* *03*	—	*29* *.4* *34*	*7.* *14* *5*	*28* *.8* *77*	—	*33* *.4* *42*	*34* *.9* *03*	*32* *.2* *11*	—	—	—
*5*	*46* *.9* *9*	*4* *1.* *5* *56*	*4* *5.* *78* *6*	*47* *.4* *95*	—	*30* *.8* *33*	*30* *.5* *79*	*30* *.2* *01*	—	*30* *.* *23* *9*	*25* *.5* *12*	*30* *.6* *83*	—	*34* *.3* *16*	*3* *5.* *48* *8*	*34* *.3* *81*	—	—	—
*6*	*47* *.7* *21*	*4* *1.* *9* *48*	*47* *.4* *06*	—	—	*3* *1.* *64* *2*	*30* *.9* *42*	*30* *.6* *03*	—	*30* *.* *64* *4*	*27* *.9* *42*	*30* *.9* *47*	—	*34* *.8* *94*	*36* *.4* *51*	*46* *.* *77* *3*	—	—	—
*7*	—	*46* *.* *25* *1*	*49* *.7* *86*	—	—	*32* *.2* *2*	*3* *1.* *35* *7*	*30* *.9* *96*	—	*3* *1.* *0* *51*	*28* *.0* *62*	*3* *1.* *59* *1*	—	*35* *.5* *05*	*37* *.7* *6*	—	—	—	—
*8*	—	*46* *.9* *47*	—	—	—	*32* *.4* *97*	*32* *.7* *41*	*32* *.4* *56*	—	*3* *1.* *2* *82*	*29* *.* *47* *5*	*32* *.* *71* *7*	—	*35* *.9* *94*	*38* *.6* *87*	—	—	—	—
*9*	—	*47* *.6* *83*	—	—	—	*32* *.9* *17*	*32* *.9* *39*	*32* *.6* *83*	—	*3* *1.* *96*	*29* *.8* *04*	*37* *.0* *67*	—	*37* *.7* *65*	*46* *.7* *4*	—	—	—	—
*10*	—	*49* *.8* *9*	—	—	—	*34* *.* *14* *4*	*33* *.* *34* *8*	*33* *.0* *85*	—	*32* *.5* *02*	*30* *.2* *88*	*37* *.3* *34*	—	*38* *.* *11* *7*	—	—	—	—	—
*11*	—	—	—	—	—	*34* *.3* *59*	*3* *5.* *13* *2*	*34* *.9* *15*	—	*33* *.* *13*	*30* *.6* *96*	*37* *.9* *6*	—	*38* *.6* *9*	—	—	—	—	—
*12*	—	—	—	—	—	*34* *.* *67* *9*	*3* *5.* *71* *8*	*35* *.5* *06*	—	*34* *.9* *54*	*3* *1.* *0* *96*	*4* *1.* *5* *58*	—	*39* *.2* *28*	—	—	—	—	—
*13*	—	—	—	—	—	*34* *.9* *23*	*36* *.6* *39*	*36* *.4* *37*	—	*3* *5.* *1* *68*	*3* *1.* *3* *34*	—	—	*46* *.2* *61*	—	—	—	—	—
*14*	—	—	—	—	—	*3* *5.* *24* *7*	*37* *.2* *59*	*37* *.1* *5*	—	*35* *.5* *5*	*32* *.0* *04*	—	—	*46* *.* *71* *2*	—	—	—	—	—
*15*	—	—	—	—	—	*36* *.* *17* *6*	*37* *.5* *04*	*37* *.3* *35*	—	*3* *5.* *7* *44*	*32* *.5* *34*	—	—	*50* *.0* *66*	—	—	—	—	—
*16*	—	—	—	—	—	*36* *.* *77* *4*	*37* *.8* *71*	*37* *.7* *24*	—	*37* *.0* *99*	*36* *.4* *94*	—	—	—	—	—	—	—	—
*17*	—	—	—	—	—	*37* *.0* *4*	*38* *.8* *57*	*38* *.7* *06*	—	*37* *.3* *72*	*37* *.0* *98*	—	—	—	—	—	—	—	—
*18*	—	—	—	—	—	*37* *.2* *73*	*4* *1.* *6* *91*	*46* *.8* *49*	—	*37* *.8* *13*	*37* *.3* *76*	—	—	—	—	—	—	—	—
*19*	—	—	—	—	—	*37* *.4* *43*	*47* *.5* *55*	*47* *.5* *69*	—	*38* *.0* *08*	*38* *.0* *02*	—	—	—	—	—	—	—	—
*20*	—	—	—	—	—	*37* *.7* *92*	—	—	—	*4* *1.* *57* *5*	*46* *.9* *23*	—	—	—	—	—	—	—	—
*21*	—	—	—	—	—	*38* *.2* *85*	—	—	—	*45* *.3* *61*	*47* *.5* *88*	—	—	—	—	—	—	—	—
*22*	—	—	—	—	—	*38* *.5* *6*	—	—	—	*46* *.* *77* *9*	—	—	—	—	—	—	—	—	—
*23*	—	—	—	—	—	*40* *.* *34* *5*	—	—	—	—	—	—	—	—	—	—	—	—	—
*24*	—	—	—	—	—	*4* *1.* *0* *09*	—	—	—	—	—	—	—	—	—	—	—	—	—
*25*	—	—	—	—	—	*47* *.0* *29*	—	—	—	—	—	—	—	—	—	—	—	—	—

Sample numbers assigned to plant extracts are *D. bulbifera *bulb extracts (1) petroleum ether, (2) ethyl acetate, (3) methanol, (4) 70% ethanol; *G. glauca* leaf extracts (5) petroleum ether, (6) ethyl acetate, (7) methanol, (8) 70% ethanol; *G. glauca *flower extracts (9) petroleum ether, (10) ethyl acetate, (11) methanol, (12) 70% ethanol; *G. glauca* stem extracts (13) petroleum ether, (14) ethyl acetate, (15) methanol, and (16) 70 % ethanol. (—) No peak.
