# Supplementary material for: Antidiabetic Activity of Gnidia glauca and Dioscorea bulbifera: Potent Amylase and Glucosidase Inhibitors
Source: Evid Based Complement Alternat Med. 2011 Jul 9;2012:929051. doi: 10.1155/2012/929051 (PMC3140190; doi:10.1155/2012/929051)
Supplement: Supplementary file 1 — Dose response curves of petroleum ether, ethyl acetate, methanol and 70% ethanol extract of D. bulbifera and G. glauca against porcine pancreatic α-amylase, crude murine pancreatic, small intestinal, liver glucosidase and α-glucosidase are available in the supplementary material. Graphical data for HPLC fingerprinting of all the extracts of D. bulbifera and G. glauca are presented here. [file 929051.f1.pdf]

Supplementary Information

Anti-diabetic Activity of *Gnidia glauca* and *Dioscorea bulbifera*: Potent Amylase and Glucosidase Inhibitors

Sougata Ghosh, [a] Mehul Ahire,[a] Sumersing Patil,[a] Amit Jabgunde, [c] Meenakshi Bhat Dusane,[a] Bimba N .Joshi, [d] Karishma Pardesi[b], Sanjay Jachak [e], Dilip D. Dhavale[c] and Balu A. Chopade [*a]

[a] *Institute of Bioinformatics and Biotechnology, University of Pune, Pune-411007, India,*

[b]*Department of Microbiology, University of Pune, Pune-411007, India,*

[c] *Garware Research Centre, Department of Chemistry, University of Pune, Pune-411007, India,*

[d]*Animal Sciences Division, Agharkar Research Institute, Pune 411 004, India,* and

[e]*National Institute of Pharmaceutical Education and Research, Mohali-160062, India*

directoribb@.unipune.ac.in

List of figures

Page(s)

Figure S1. Dose response curve of petroleum ether extracts against porcine pancreatic α-amylase. S3

Figure S2. Dose response curve of ethyl acetate extracts against porcine pancreatic α-amylase. S4

Figure S3. Dose response curve of methanol extracts against porcine pancreatic α-amylase. S5

Figure S4. Dose response curve of 70% ethanol extracts against porcine pancreatic α-amylase. S6

Figure S5. Dose response curve of petroleum ether extracts against murine S7

pancreatic glucosidase.

Figure S6. Dose response curve of ethyl acetate extracts against murine pancreatic glucosidase. S8

Figure S7. Dose response curve of methanol extracts against murine pancreatic glucosidase. S9

Figure S8. Dose response curve of 70 % ethanol extracts against murine pancreatic glucosidase. S10

Figure S9. Dose response curve of petroleum ether extracts against murine small intestinal glucosidase. S11

Figure S10. Dose response curve of ethyl acetate extracts against murine small intestinal glucosidase. S12

Figure S11. Dose response curve of methanol extracts against murine small intestinal glucosidase. S13

Figure S12. Dose response curve of 70 % ethanol extracts against murine small intestinal glucosidase. S14

Figure S13.Dose response curve of petroleum ether extracts against murine liver glucosidase. S15

Figure S14. Dose response curve of ethyl acetate extracts against murine liver glucosidase. S16

Figure S15. Dose response curve of methanol extracts against murine liver glucosidase. S17

Figure S16. Dose response curve of 70 % ethanol extracts against murine liver glucosidase. S18

Figure S17. Dose response curve of petroleum ether extracts against α- glucosidase. S19

Figure S18. Dose response curve of ethyl acetate extracts against α- glucosidase. S20

Figure S19. Dose response curve of methanol extracts against α- glucosidase. S21

Figure S20. Dose response curve of 70 % ethanol extracts against α- glucosidase. S22

Figure S21. HPLC fingerprint of *D. bulbifera* bulb (A) petroleum ether (B) S23

ethyl acetate (C) methanol (D) 70 % ethanol extract compared with chromatogram of a standard solution containing three marker compounds.

Figure S22. HPLC fingerprint of *G. glauca* leaf (A) petroleum ether (B) ethyl acetate (C) methanol (D) 70 % ethanol extract compared with chromatogram of a standard solution containing three marker compounds. S23

Figure S23. HPLC fingerprint of *G. glauca* flower (A) petroleum ether (B) ethyl acetate (C) methanol (D) 70 % ethanol extract compared with chromatogram of a standard solution containing three marker compounds. S24

Figure S24. HPLC fingerprint of *G. glauca* stem (A) petroleum ether (B) ethyl acetate (C) methanol (D) 70 % ethanol extract compared with chromatogram of a standard solution containing three marker compounds. S25

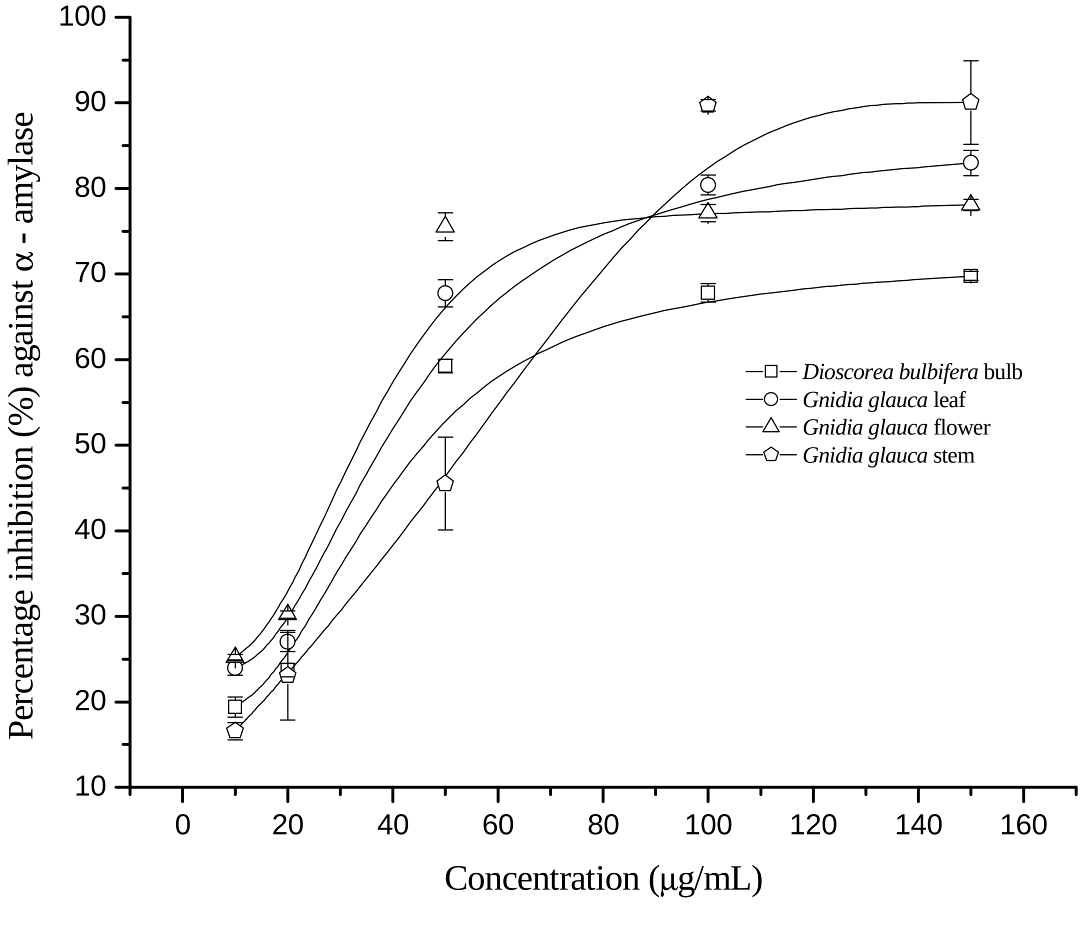

Figure S1. Dose response curve of petroleum ether extracts against porcine pancreatic α-amylase.

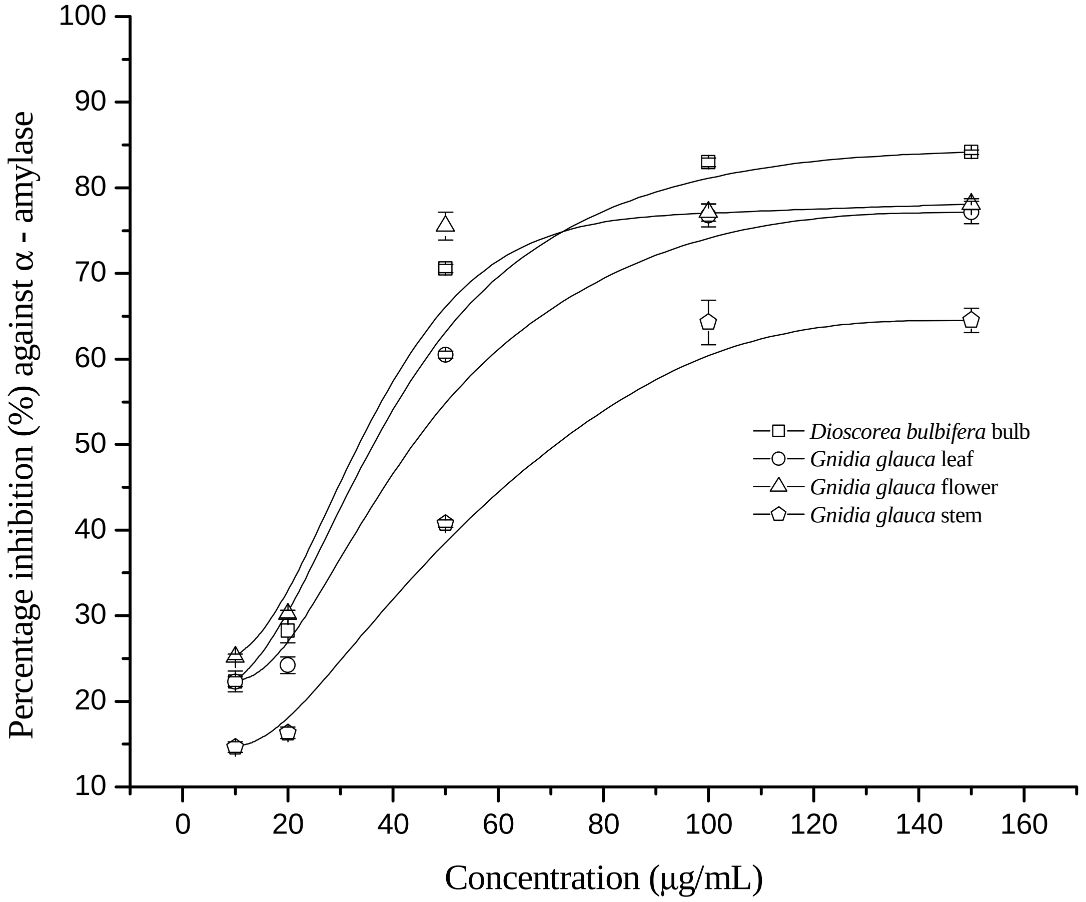

Figure S2. Dose response curve of ethyl acetate extracts against porcine pancreatic α-amylase.

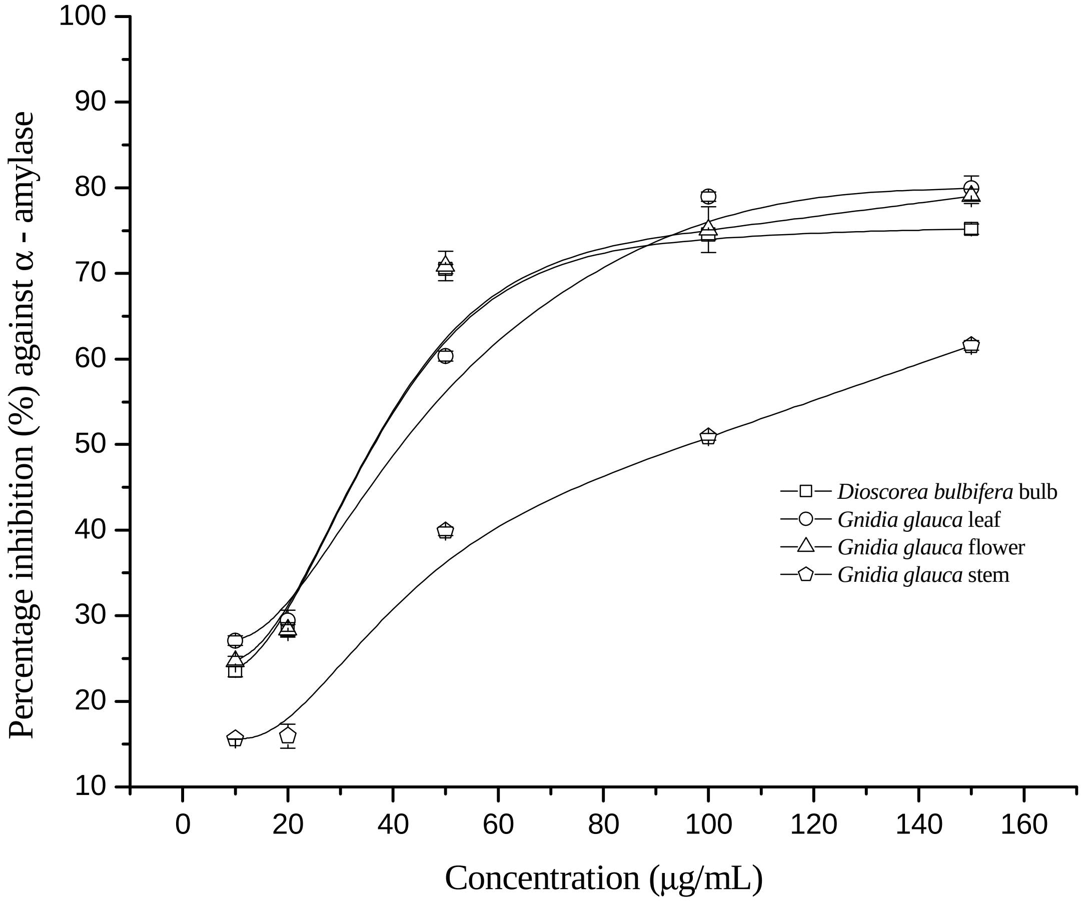

Figure S3. Dose response curve of methanol extracts against porcine pancreatic α-amylase.

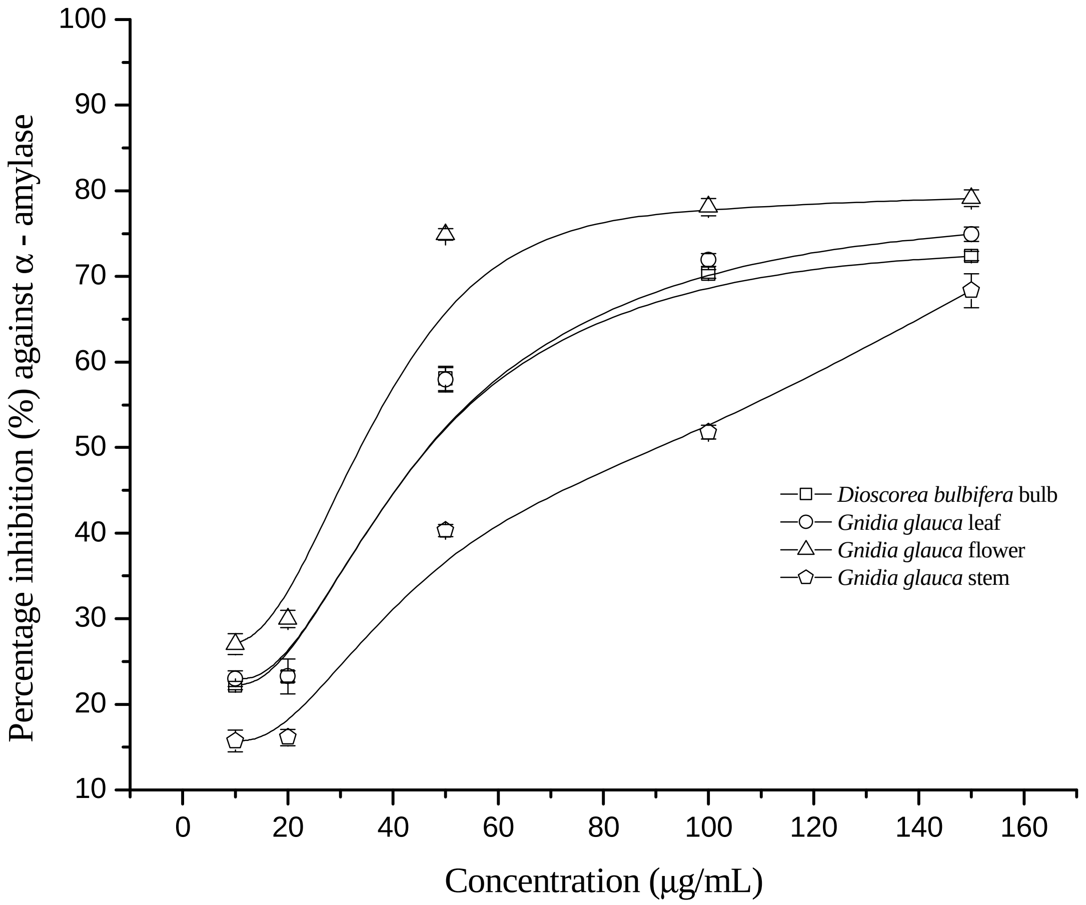

Figure S4. Dose response curve of 70% ethanol extracts against porcine pancreatic α-amylase.

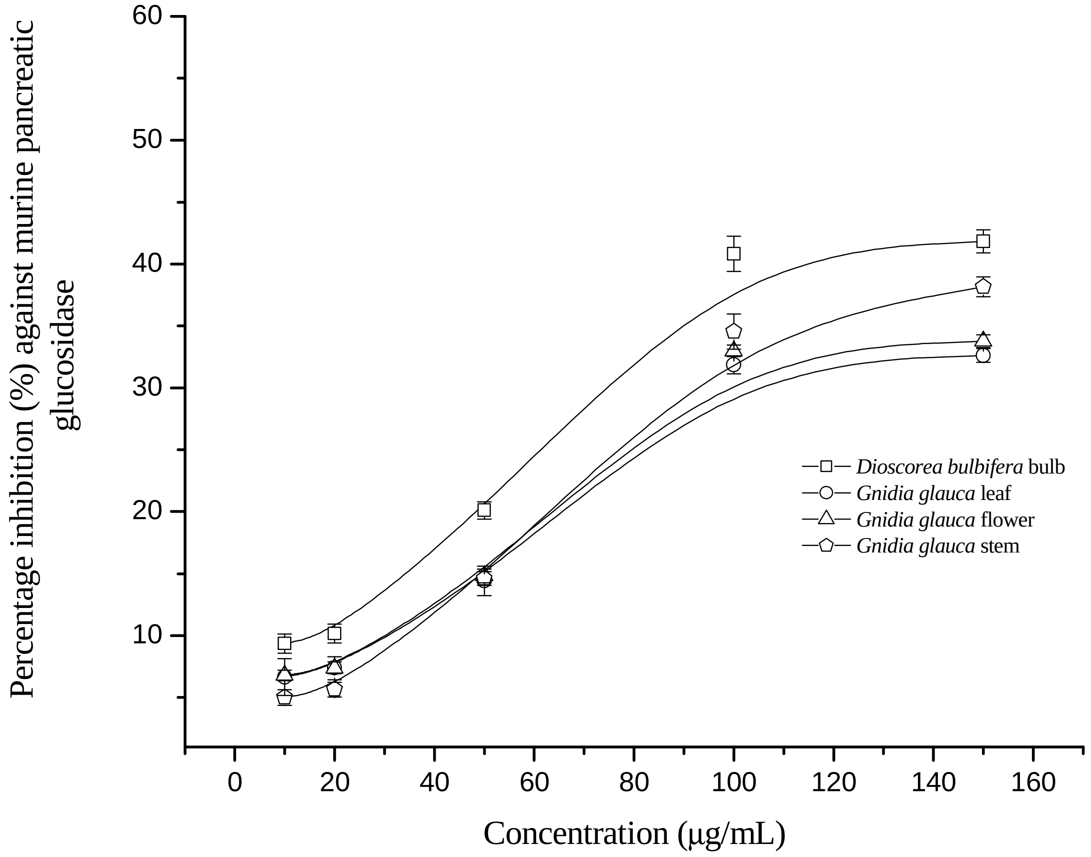

Figure S5. Dose response curve of petroleum ether extracts against murine pancreatic glucosidase.

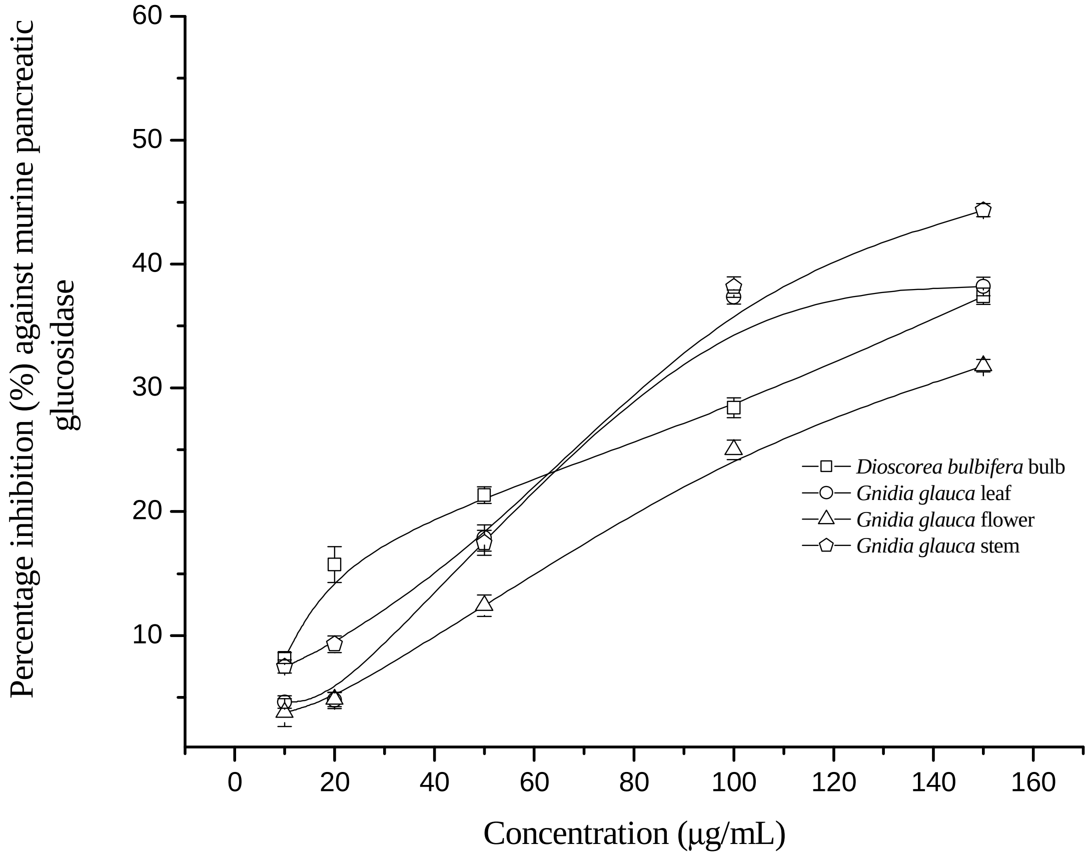

Figure S6. Dose response curve of ethyl acetate extracts against murine pancreatic glucosidase.

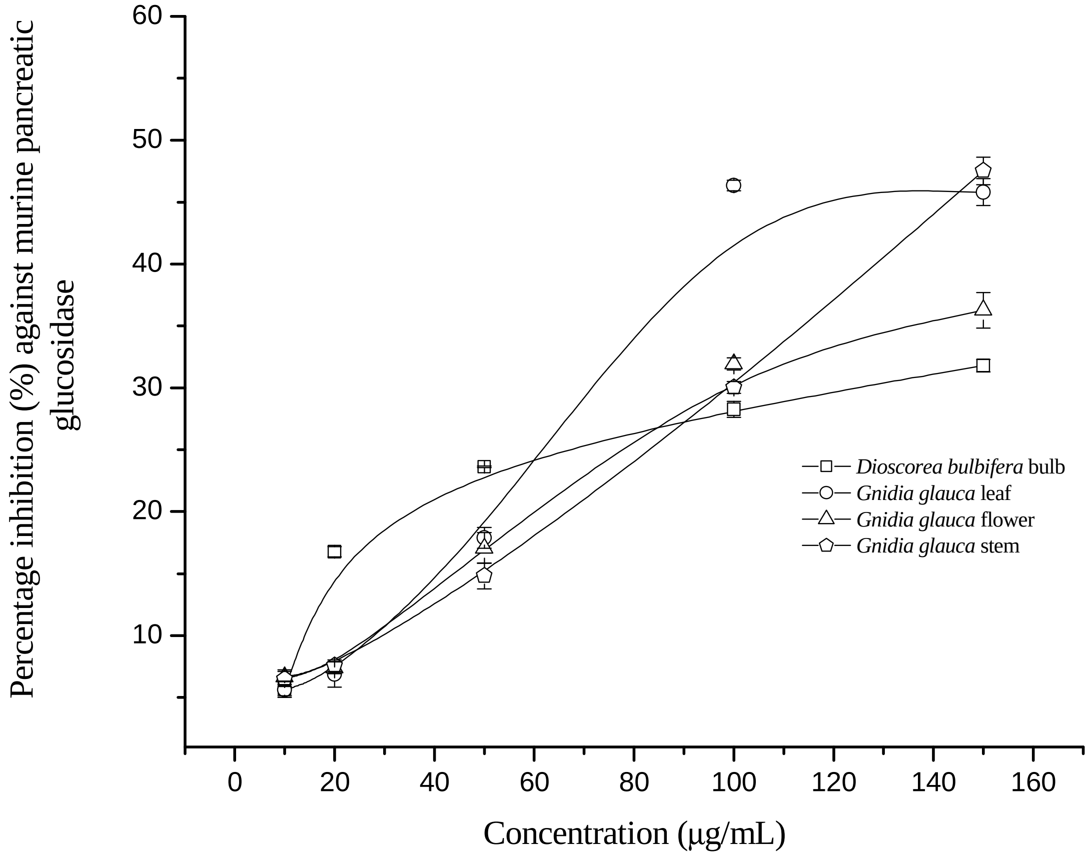

Figure S7. Dose response curve of methanol extracts against murine pancreatic glucosidase.

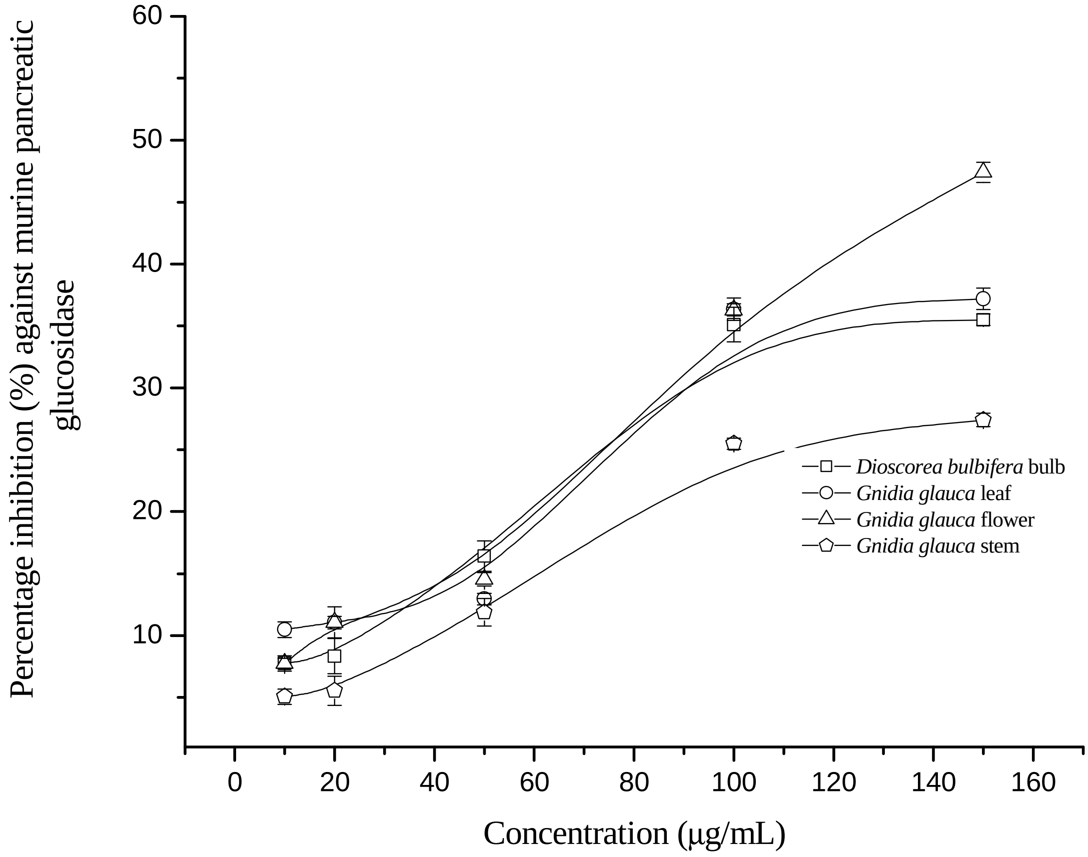

Figure S8. Dose response curve of 70 % ethanol extracts against murine pancreatic glucosidase.

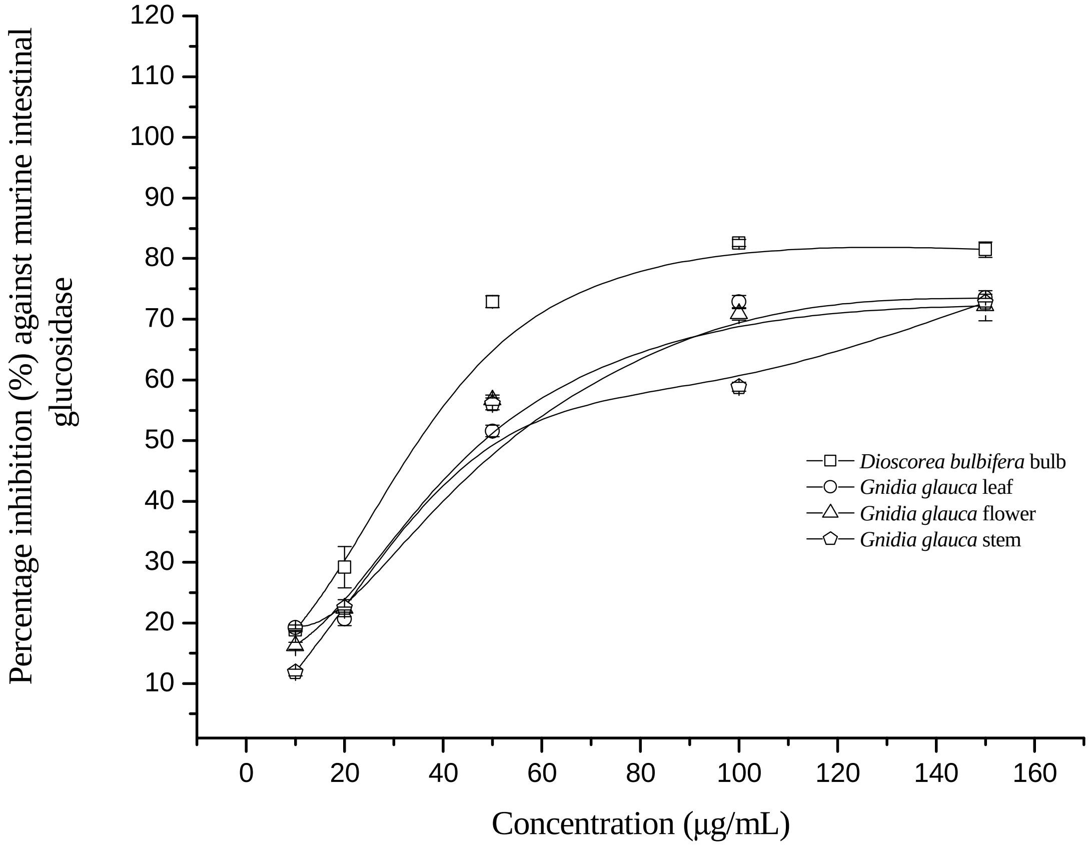

Figure S9. Dose response curve of petroleum ether extracts against murine small intestinal glucosidase.

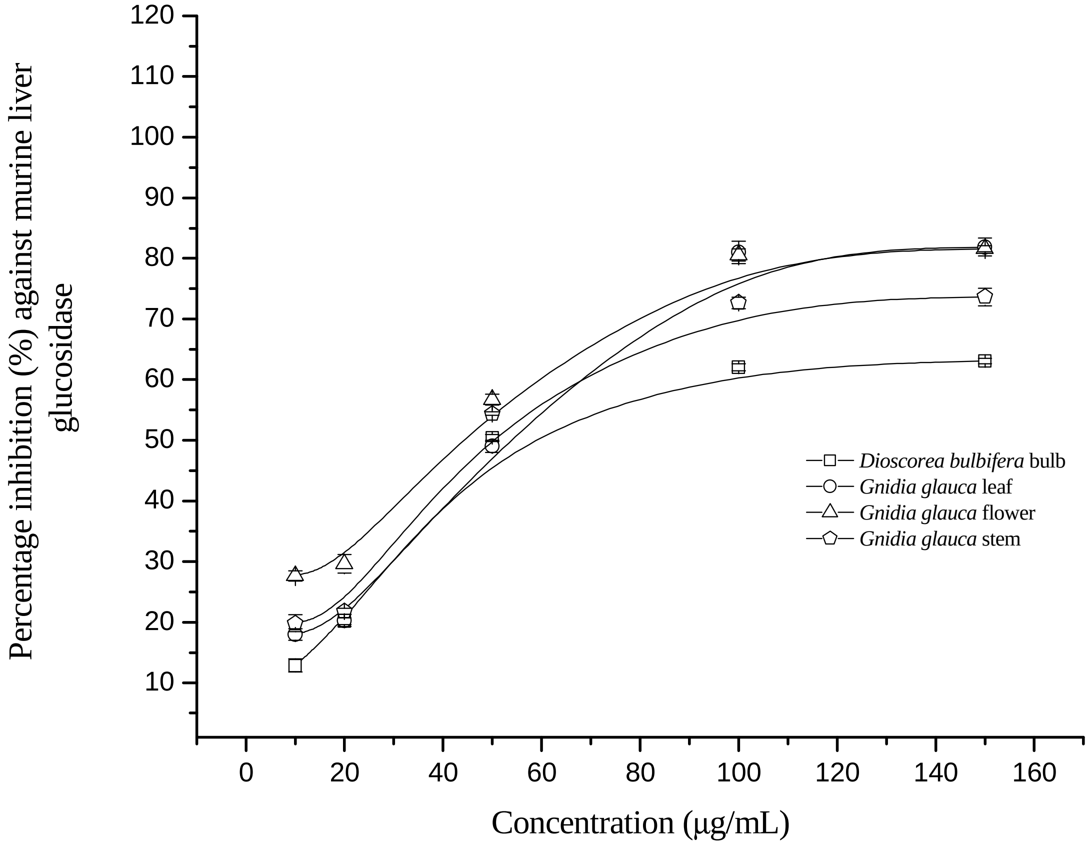

Figure S10. Dose response curve of ethyl acetate extracts against murine small intestinal glucosidase.

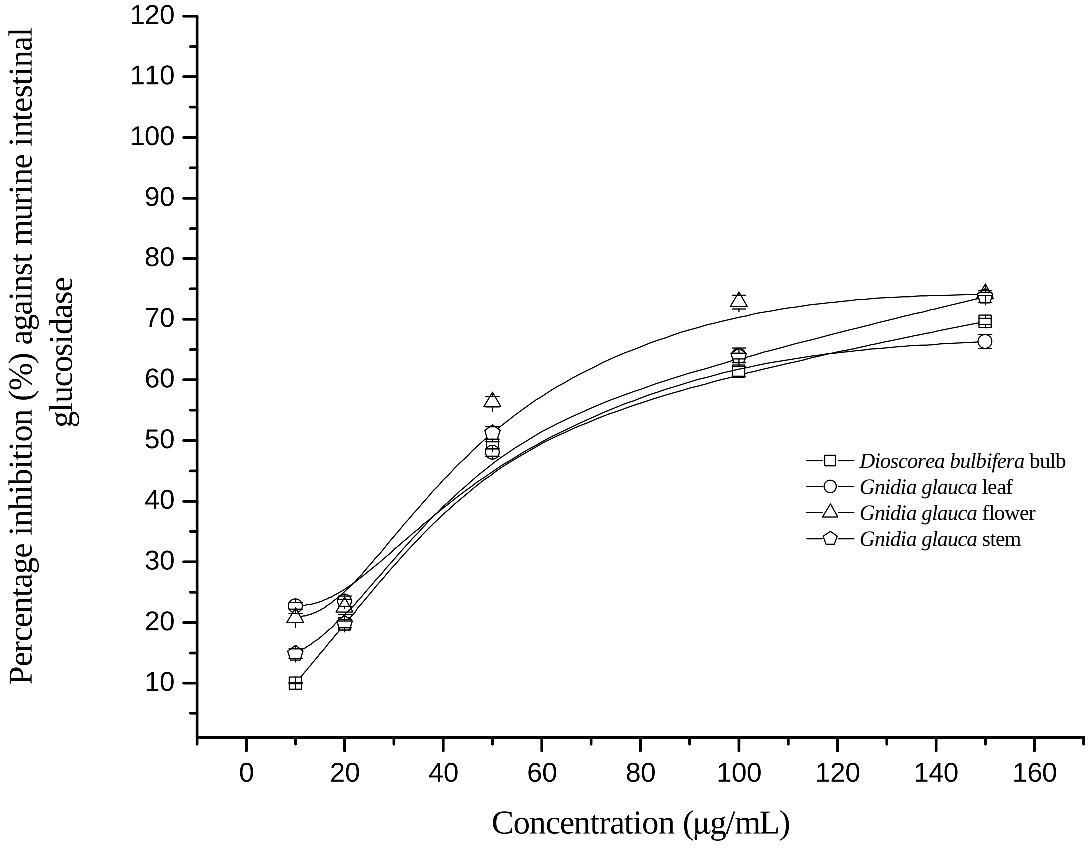

Figure S11. Dose response curve of methanol extracts against murine small intestinal glucosidase.

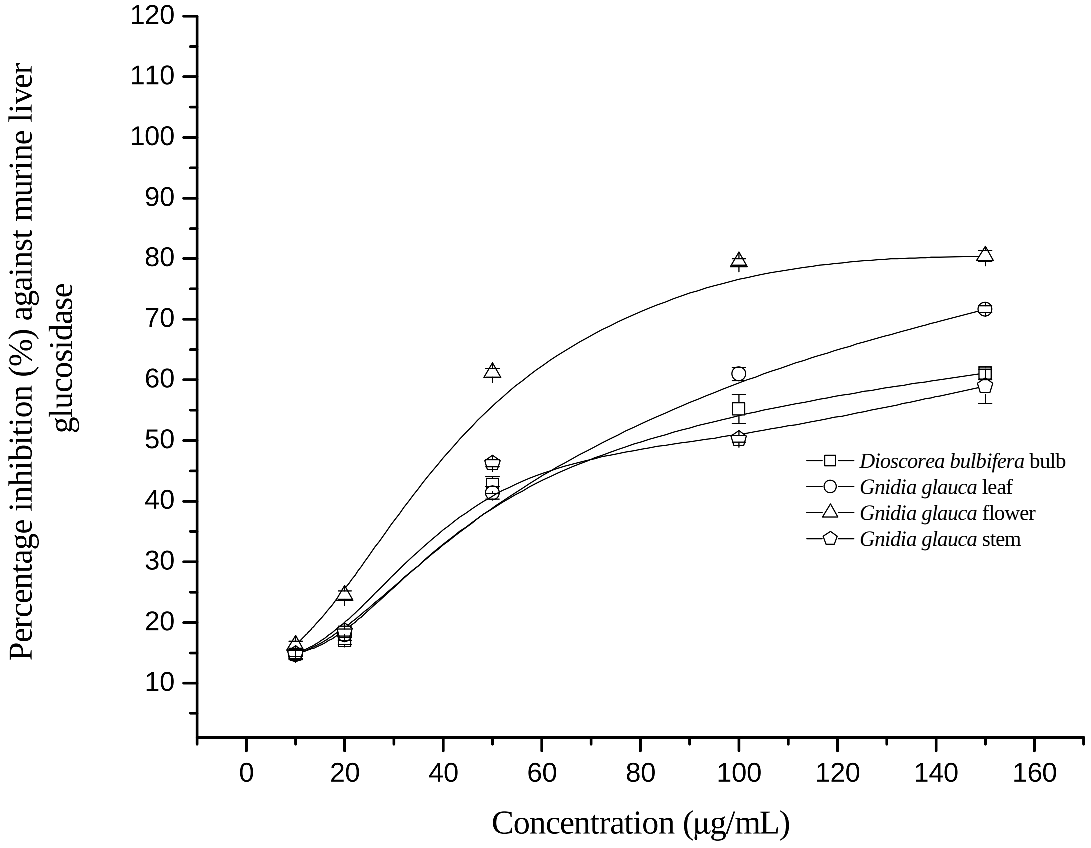

Figure S12. Dose response curve of 70 % ethanol extracts against murine small intestinal glucosidase.

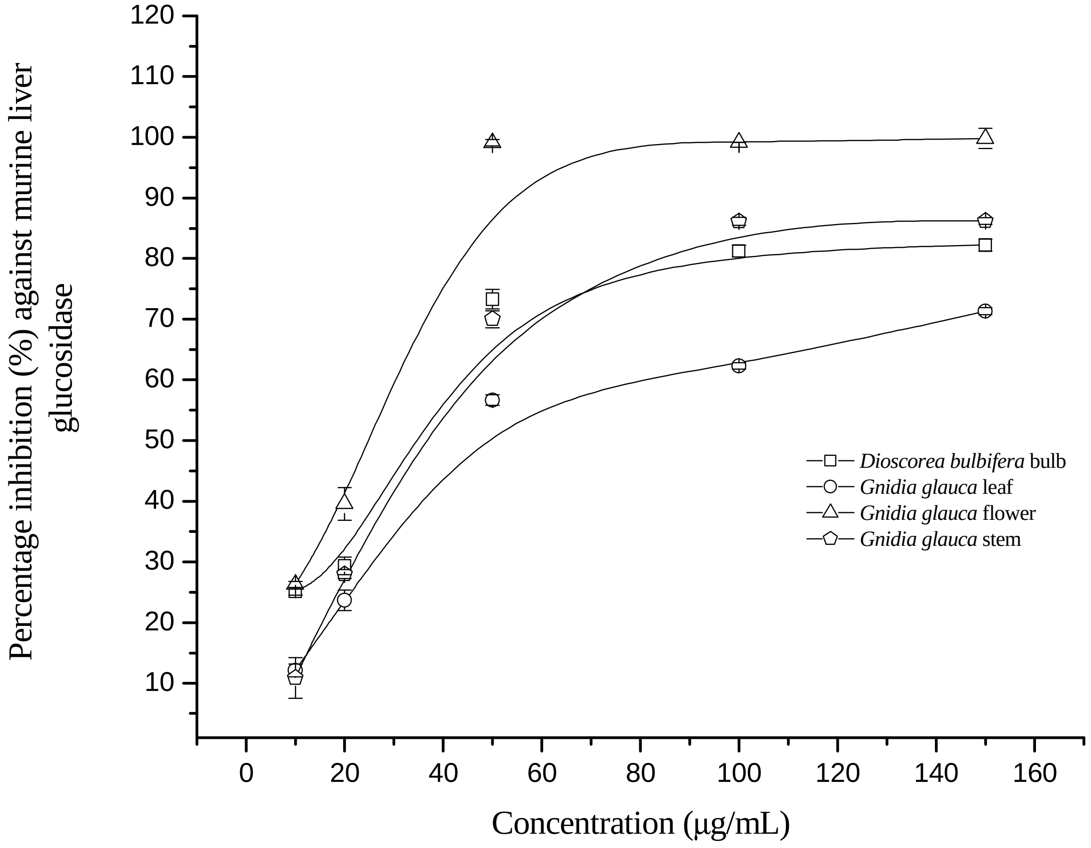

Figure S13. Dose response curve of petroleum ether extracts against murine liver glucosidase.

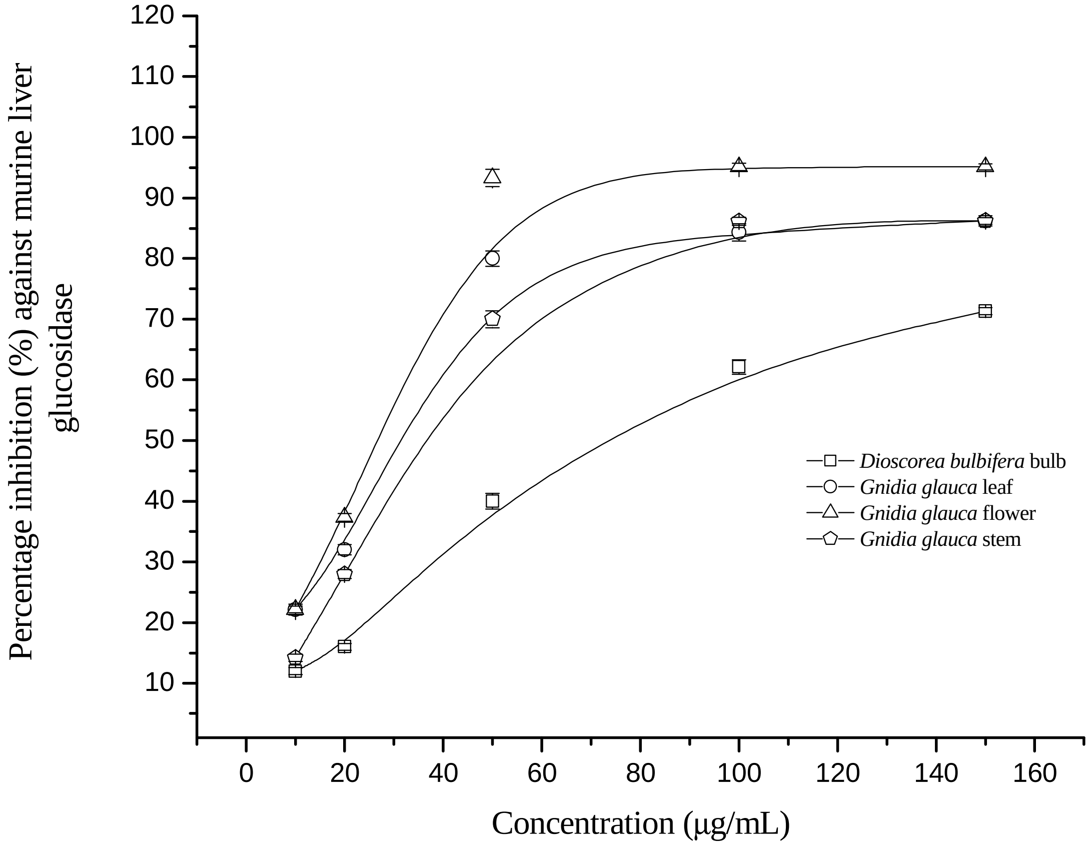

Figure S14. Dose response curve of ethyl acetate extracts against murine liver glucosidase.

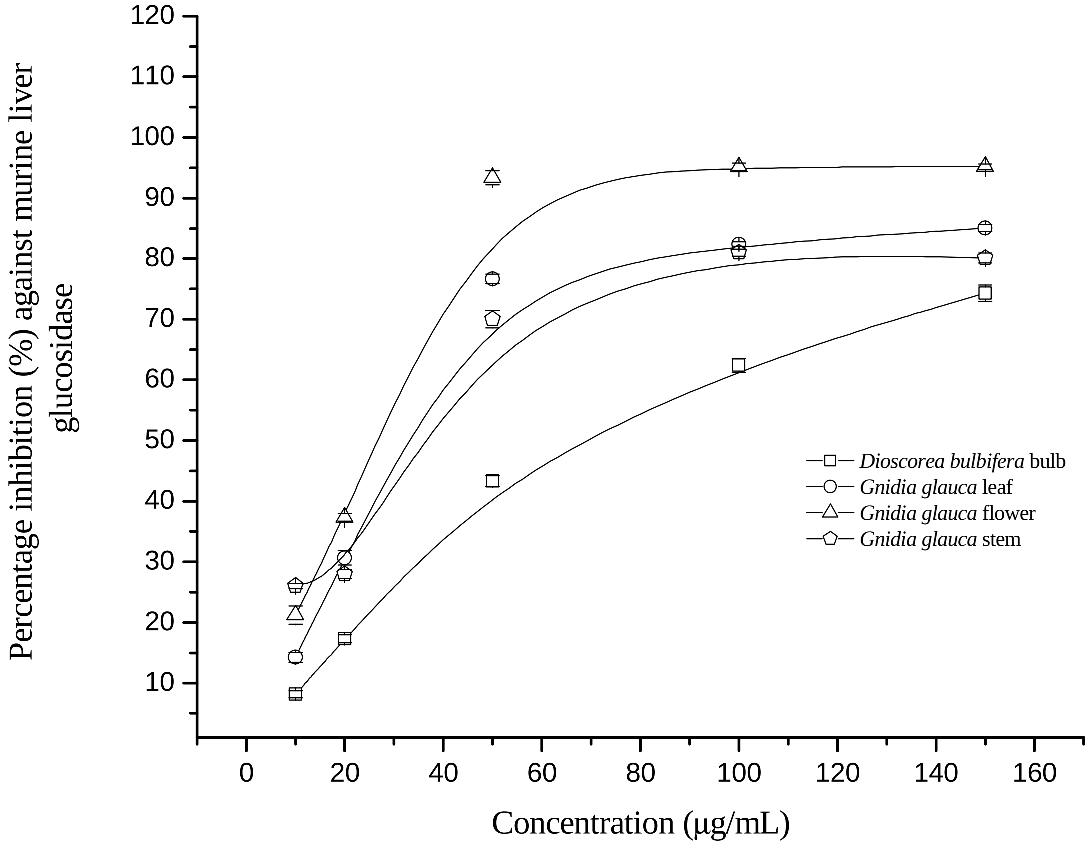

Figure S15. Dose response curve of methanol extracts against murine liver glucosidase.

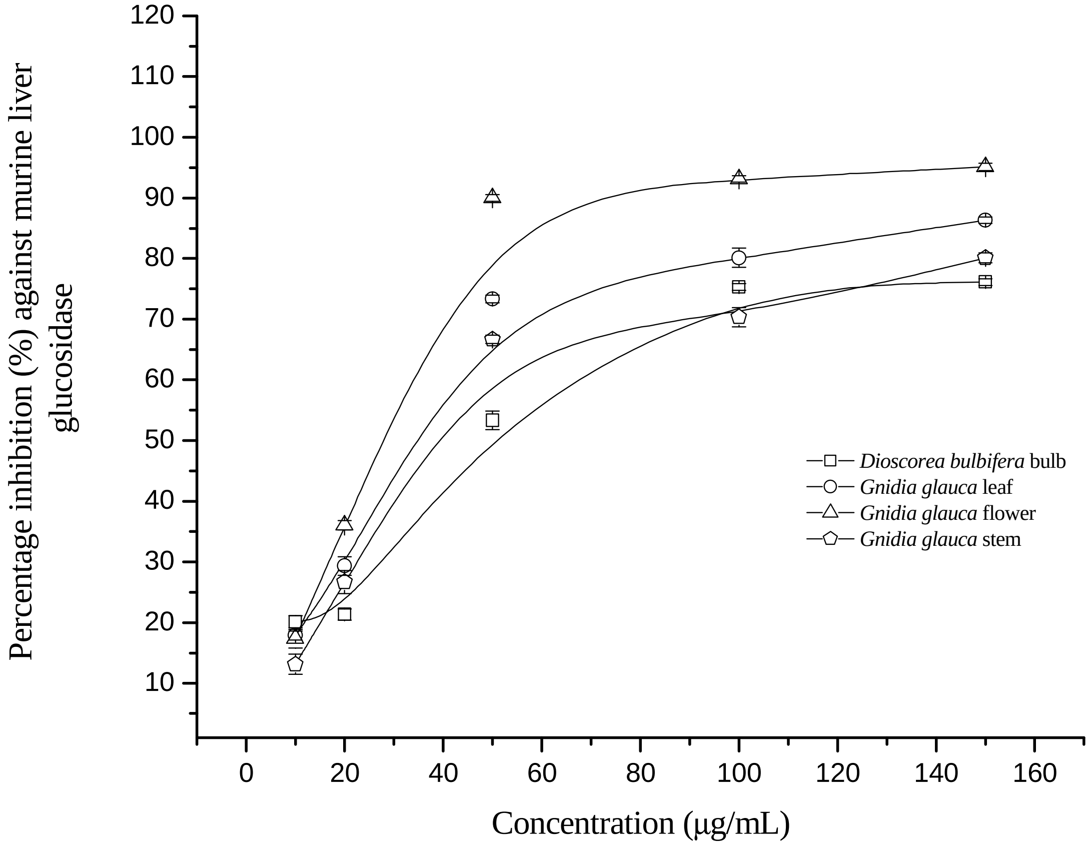

Figure S16. Dose response curve of 70 % ethanol extracts against murine liver glucosidase.

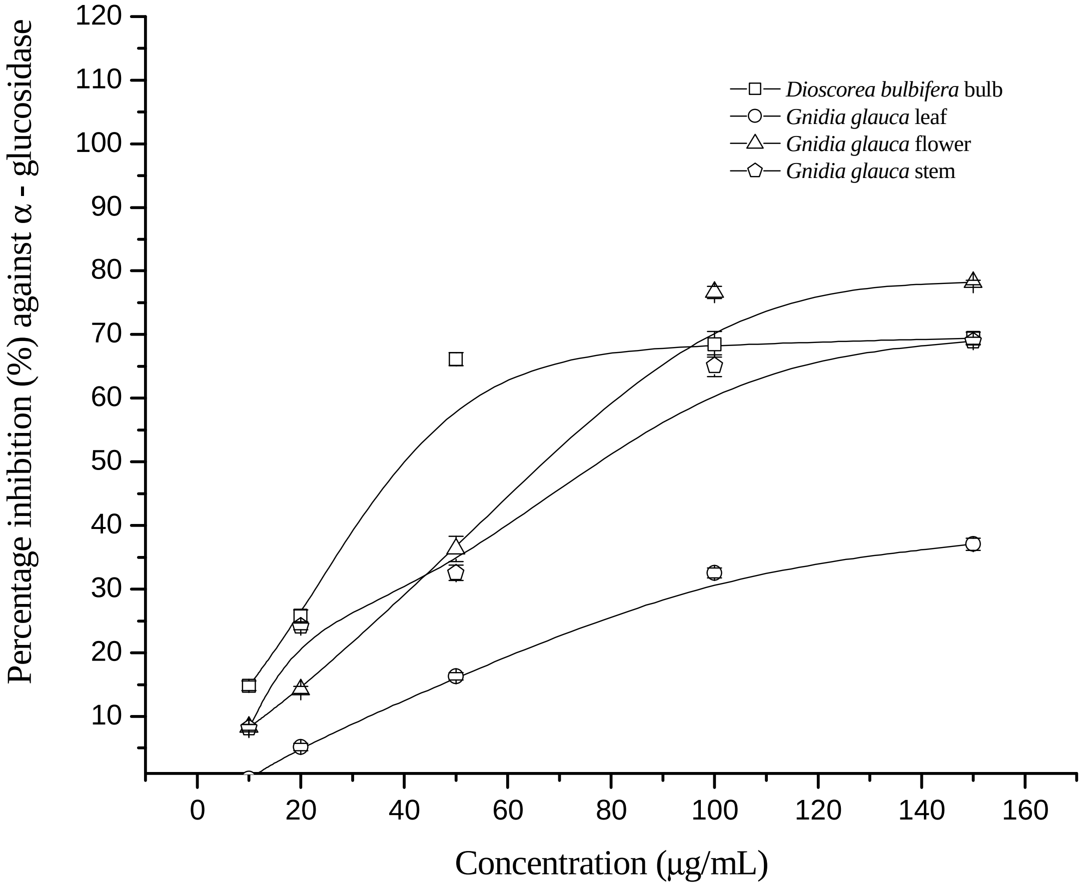

Figure S17. Dose response curve of petroleum ether extracts against α- glucosidase.

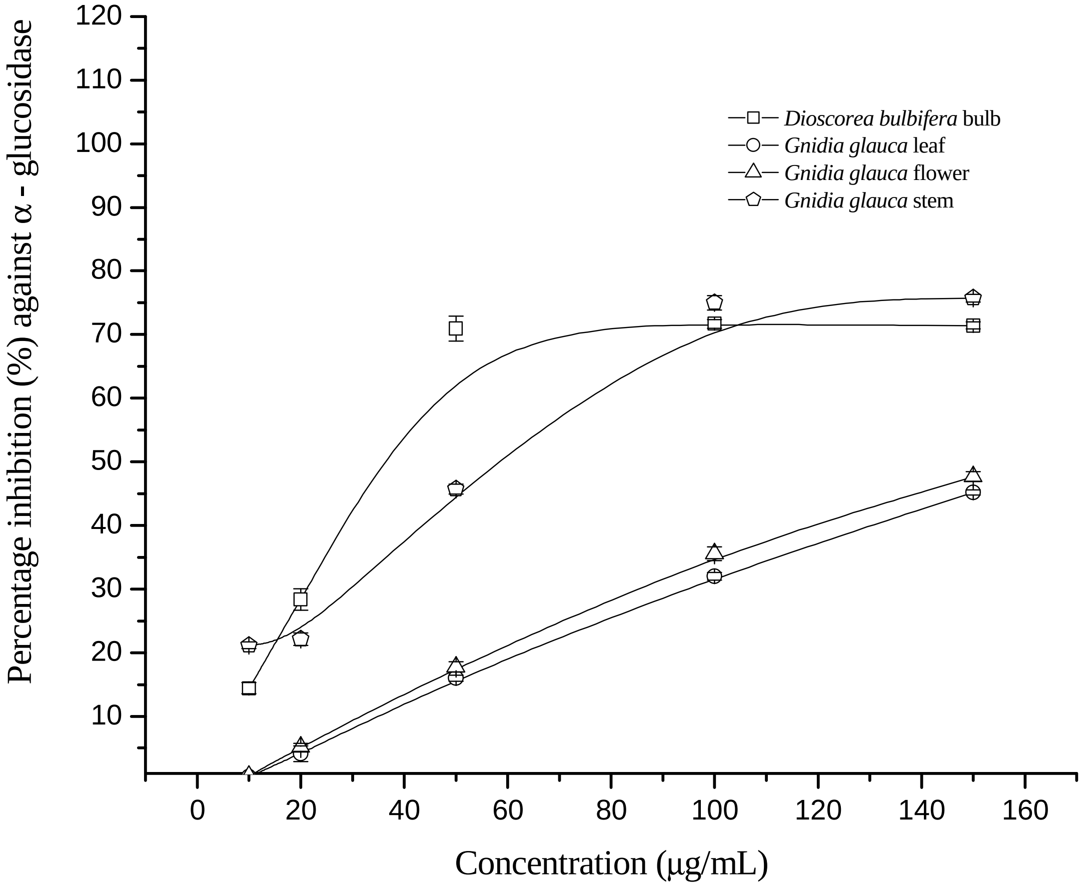

Figure S18. Dose response curve of ethyl acetate extracts against α- glucosidase.

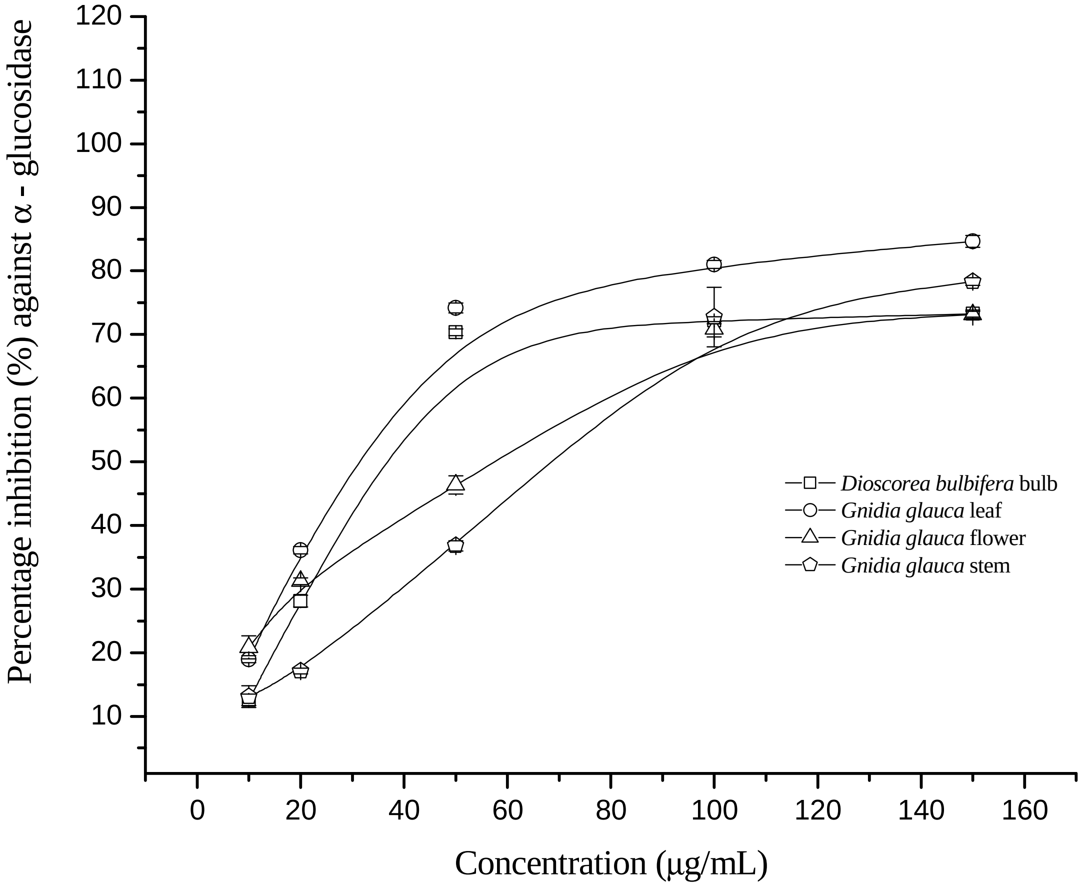

Figure S19. Dose response curve of methanol extracts against α- glucosidase.

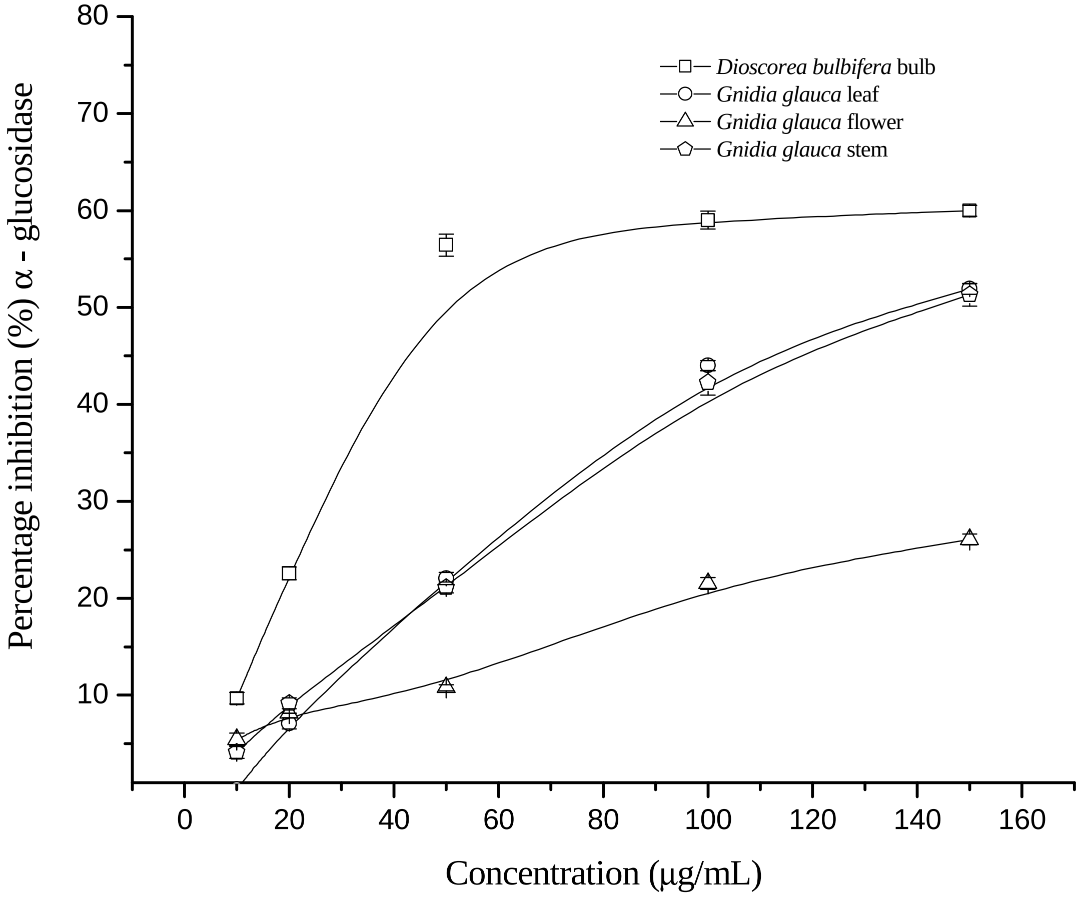

Figure S20. Dose response curve of 70 % ethanol extracts against α- glucosidase.

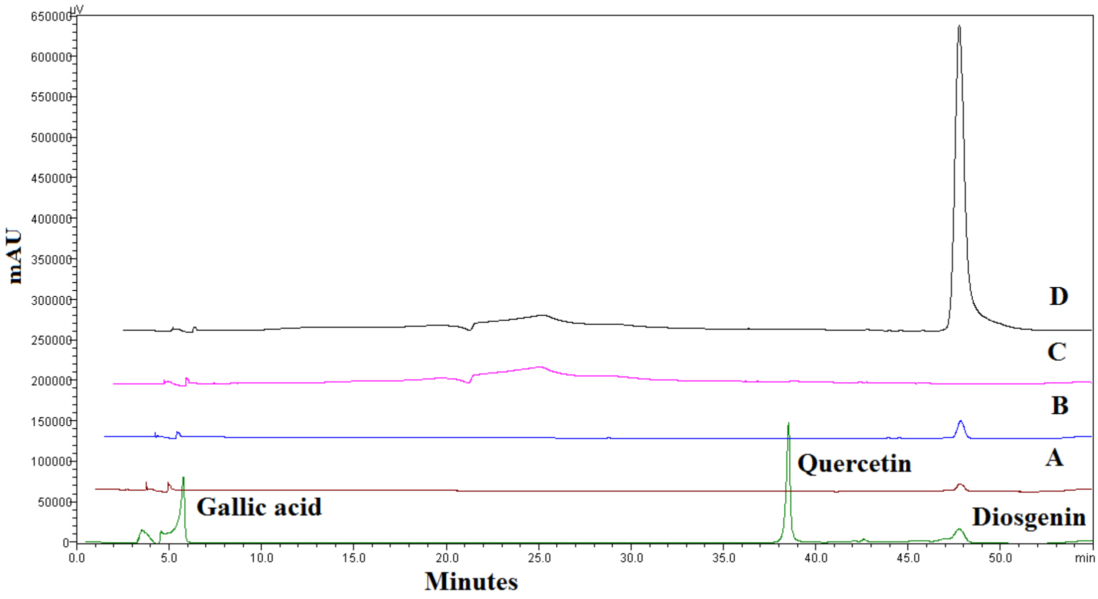

Figure S21. HPLC fingerprint of *D. bulbifera* bulb (A) petroleum ether (B) ethyl acetate (C) methanol (D) 70 % ethanol extract compared with chromatogram of a standard solution containing three marker compounds.

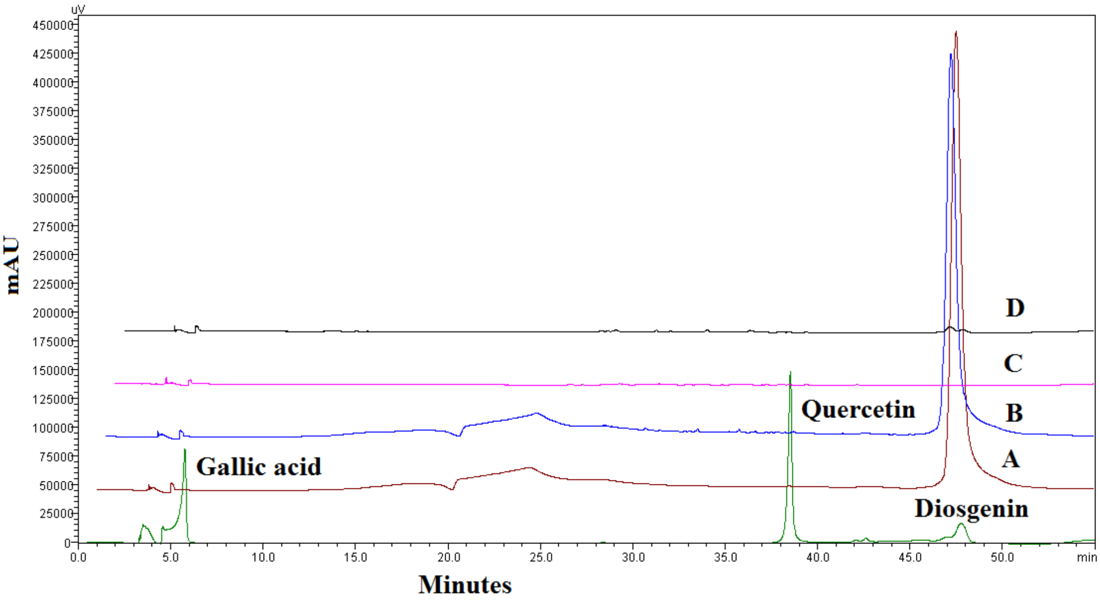

Figure S22. HPLC fingerprint of *G. glauca* leaf (A) petroleum ether (B) ethyl acetate (C) methanol (D) 70 % ethanol extract compared with chromatogram of a standard solution containing three marker compounds.

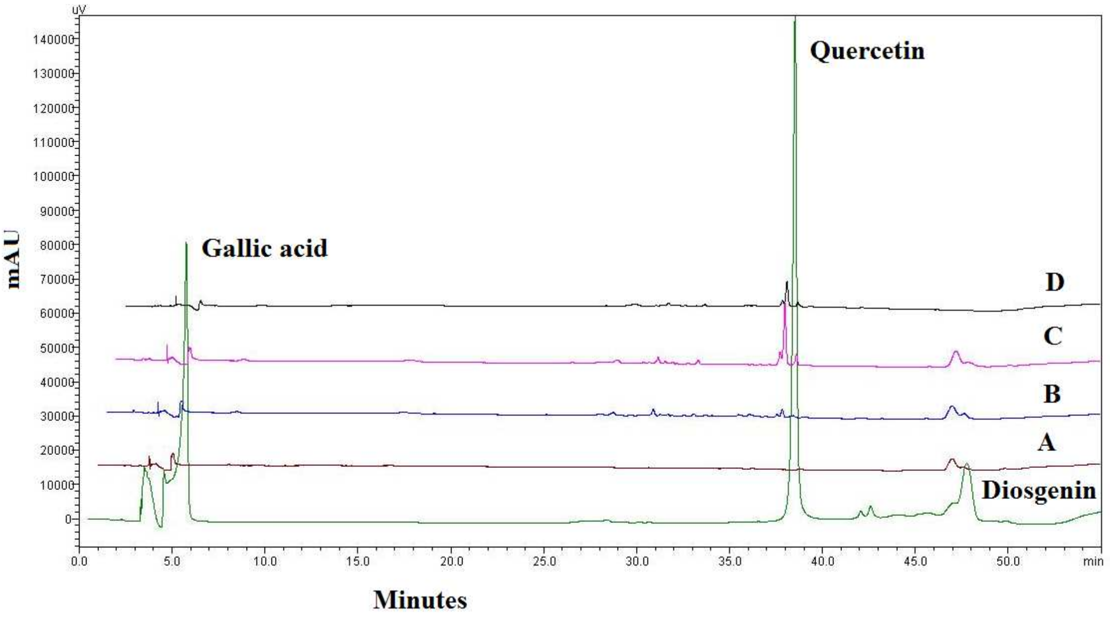

Figure S23. HPLC fingerprint of *G. glauca* flower (A) petroleum ether (B) ethyl acetate (C) methanol (D) 70 % ethanol extract compared with chromatogram of a standard solution containing three marker compounds.

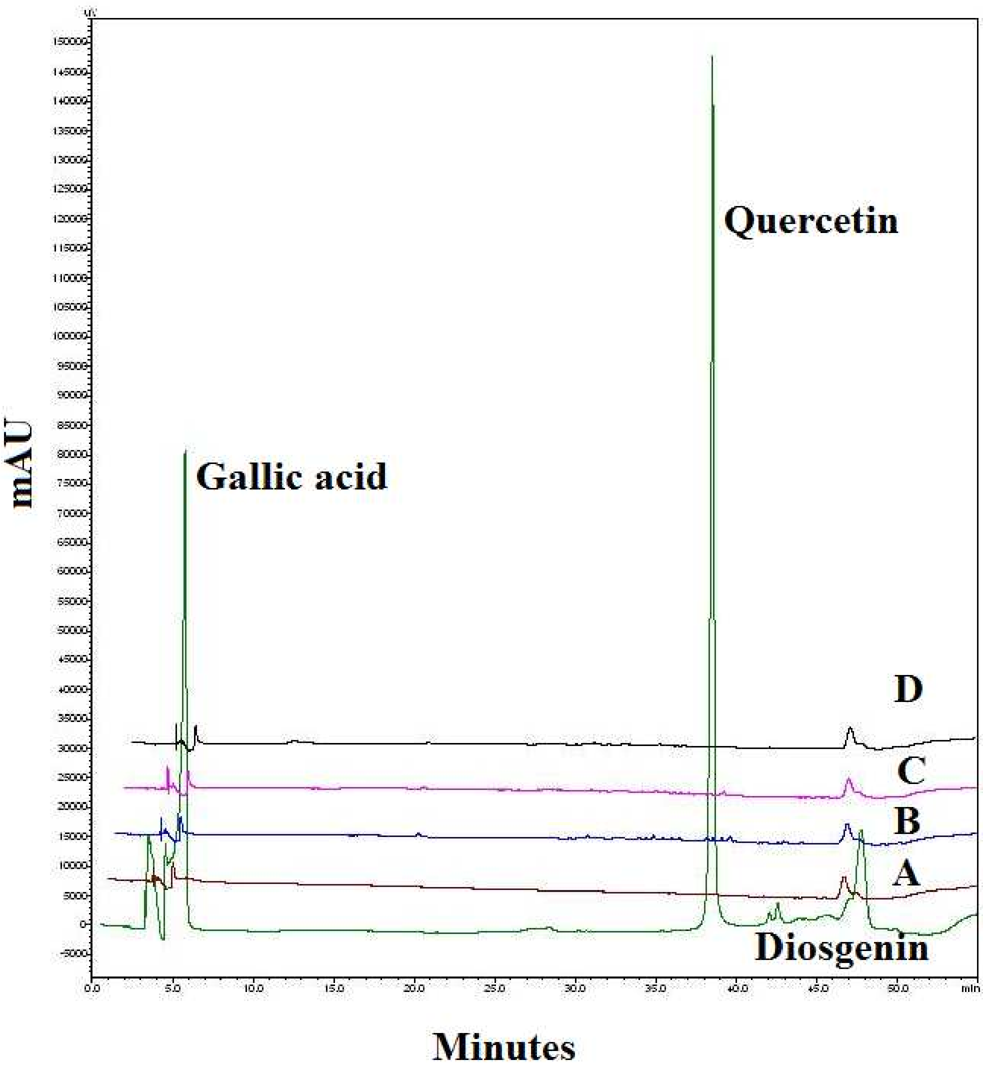

Figure S24. HPLC fingerprint of *G. glauca* stem (A) petroleum ether (B) ethyl acetate (C) methanol (D) 70 % ethanol extract compared with chromatogram of a standard solution containing three marker compounds.
